# Quarterly Periscope

**Published:** 1823-06-01

**Authors:** 


### Quarterly Periscope

OF

### PRACTICAL MEDICINE;

BEING THE

### Spirit of the Public Journals,

FOREIGN AND DOMESTIC.

*Paucis libris immorari et innutriri oportet, si velis aliquid trahere, quod in animo fideliter hæreat.* SENECA.

Duo vitia vitanda sunt in cognitionis et scientiæ studio. ***** Alterum est vitium, quod quidam nimis magnam operam conferunt in res obscuras atque difficiles, easdemque non necessarias. CICERO.

1. *Observations on the Dracunculus, or Guinea-worm.* By the late DR. HELENUS SCOTT, of Bombay.

[Communicated to Dr. Johnson on the Author's departure for New South Wales, 1822.]

THE Dracunculus or Guinea Worm deserves to be better known, for it gives rise to very important and very distressing effects in tropical climates. A thousand conjectures, and some of them very absurd, have arisen with regard to the natural history of this animal. Some writers deny its vitality, and a very recent author and well known physician has said, that it is nothing but an inanimate prolongation of the cutis, like the string of a fiddle in its appearance, and in some of its properties. This is very entertaining!

A medical gentleman, a very few years ago, on the Malabar Coast, looking at a gardener digging the ground, saw him turn up a quantity of something like the hairs of a horse. On inquiring what it was, for it excited no curiosity nor attention in the Indian, he was told that it was a quantity of Guinea-worms, and that during the rainy season they often found them in this state in the wet earth. He collected a considerable number of them, a sufficient quantity to fill a phial, into which he put them with alkohol. In this state he showed them to many, and gave a portion of the mass to a particular friend of mine, who is now no more. This occurrence took place soon after I left India, so that I never saw any of those that were found on this occasion, but I have as firm a conviction of the fact as I should have had if I myself had witnessed every part of the discovery. That those animals by which the human body is so often infested are bred in the moist earth there can now no longer be a doubt.

I had long known that the Guinea-worms become very abundant during the rainy season. The Indians who walk with their feet and legs bare are at this time sadly infested by this animal, That the ova from which it arises, or the young worms in a very minute form live in water is probable from the circumstance, that whenever the human skin is exposed to be wetted at this season, the Guinea-worm is soon found to effect a lodgement, and to give rise to all the symptoms of the well known disease. It may be asked how it happens that those minute worms are so seldom seen in the state of those dug up by the gardener. To this I can only reply, that perhaps the changes from a very minute form to one much more sensible may require but very little time, before the animals quit their subterraneous abode. Again I may observe, that Europeans are but very little employed in India in digging the ground, and especially during the rainy season, at which period only, those animals are in a visible state. Let me add to all this, that to make such an observation, requires a degree of attention that every man does not possess; but the natives who alone meet with it often, seem well acquainted with the fact. It is well known that the men who in India are employed in camps or elsewhere to carry water in leathern bags on their backs, are infested by this animal over all that part of the skin that has often been wetted. From all this I conclude, that while yet in a minute, perhaps an invisible state, this animal resides in a moist soil, or in water, and that coming into contact with the human skin, it adheres to it, and makes its way to the cellular membrane, which is the proper place of its residence. Wherever this membrane is diffused the worm finds its way. It generally lies just below the true skin, but it often goes far between the muscles, and I have known it residing very deep in the socket of the eyes, and without doing any material harm. I have known it take up its abode in the mouth, in the cheeks or below the tongue. It often inhabits the scrotum, and in short wherever the cellular membrane extends the dracunculus finds a home.

There cannot be a doubt but that it has some locomotive power, though we never observe their motion after extraction. The first mark of the existence of a Guinea-worm is frequently a small blister on some part of the skin, like the appearance of the cuticle from the application of blistering plaster. If this little vesicle be broken from being rubbed, it often happens that the most insufferable itching is produced over the whole body. The animal seems, on its first exposure to the air, to be irritated, and to shed a poison by which the whole system is instantly affected. This does not happen in every case, nor does it twice take place from the same worm. I can never forget the sufferings that I experienced from this cause. Little thinking that I had a Guinea-worm under the skin, I left my house one morning, when I soon became extremely sick at stomach. This was followed or combined with an intolerable itching over almost every part of the skin. I soon resolved to go into a gentleman's house, about two miles from my own, and although very near it, I could no longer have patience, but tore off almost all my clothes, to be able the better to relieve this insufferable sensation. In this condition I left the carriage, and ran into the house in a state of misery and impatience that I cannot describe. I believe I excited considerable alarm, and I think not without reason, for I had much the appearance and many of the feelings of a madman. I soon however was supplied with a tub of water, and getting two or three men to wet and rub my skin wherever I desired them, my feelings became more moderate. In an hour or two I was able to return to my own house, but the sickness of stomach and the itching did not quite leave me till the next day. I then saw and could take hold of the end of the worm several inches of which I extracted with great ease.

I must now observe, that this animal has not the power of returning into the skin from which it has been drawn, not even in the smallest degree. It is elastic, so that on being stretched it may be drawn to a greater length like a thread made of worsted. This elasticity gives it the appearance of pulling itself backwards and of returning to its former residence. I am quite sure however, that it does not possess such a power even in the least degree as I have just said. To how many errors may a wrong observation give rise? The fear of losing the worm by its retreating backwards, has given birth to much injurious practice and needless alarm. To prevent this retreat, it has been recommended to roll it on a quill, securing it with a thread, or to tie it in some other way that the prisoner may no longer be able to escape. The consequences of this tying and stretching and pulling are very hurtful in many cases, and always useless or needless. Although this animal appears so inert, and so insensible it suffers from such treatment, and produces great suffering to the patient. On first being stretched and tied it no doubt finds itself uneasy, and it has the power of communicating great uneasiness to the patient, for soon after he becomes restless and feverish. The limb becomes inflamed and painful, and all this from causes that we cannot see. I have supposed that in its efforts to relieve itself it is agitated through its whole length, and becomes the means of exciting fever and much mischief. The best means of relieving this state of suffering, is to remove all the tyings and to apply a soft poultice to the part.

The native surgeons in India have a method of extracting the Guinea-worm which often answers very well; for a worm, even when of the length of three or four feet, is sometimes extracted in a few minutes. When a worm is known to exist in any limb, they endeavour to discover the way in which it is placed. They can often feel its convolutions below the skin like a vein encircling the limb, or branching in various directions. They then raise the skin with a needle or pointed instrument, cutting off little portions of it till they have come to the animal below it. They through this well or opening introduce a probe or blunt instrument below the animal now laid bare, and getting hold of it double, they are often able to extract the whole of it at once. The success of this operation however much depends on the worm being placed in a soft or fleshy part, for if it get near the fingers or toes, or so close to any bones that its convolutions surround the bones, it is evident that it cannot be extracted without waiting perhaps for many days, till it is disposed of itself to relinquish its strong hold.

It has been thought that some means may be used to kill this worm without extracting it, an opinion that has given birth to a good deal of quackery. With such a view, I have known them use a variety of applications. I have seen the limb rubbed with mercurial ointment, and I have known a variety of vegetable poultices applied. They often take one of the thick and juicy leaves of the plant of the aloe succotorina, which is very common in India. They slit it open, and cutting it into a proper length they roast it over the fire, and apply it like a poultice to the part where the worm has appeared. This plant contains a very bitter juice, which I suppose was expected to be much disliked by the worm. It certainly makes a convenient and agreeable poultice in this way, and I have on some occasions supposed that it answered better than any thing else of that kind.

There are very many cases in which we cannot venture to make an opening in the skin for the extraction of the worm, or where it would not succeed. In such cases, it was my practice of late years, as soon as the worm had come out far enough to enable us to seize it, to pull it gently as long as it followed without resistance. I then with a pair of scissars cut off the whole of what had been extracted, leaving half or a quarter of an inch of it only. Over this a poultice or something soft was applied, and the part was tied up so as to defend the animal from farther injury. In this way I have succeeded in extracting the whole of the worm, never pulling it when I found any resistance, but bringing out as much as would readily come and cutting off daily what I had extracted, as at first. From this mode I have never seen any harm whatever to follow. All irritation is avoided, for the animal *seems* not to suffer from being cut in two, although it does from being stretched. The end of it is never long lost, for it continues to advance and make its appearance evidently retaining life and the power of motion. I have not unfrequently known the worm die in its subcuticular abode giving rise to inflammation followed by abscess with a discharge of pus and the corrupted animal. If the worm make at first its appearance near to the foot or the hand, the case is often tedious and difficult in whatever way we treat it. The worm surrounds the bones so that we must break it, but cannot extract it. The best method is to be patient, for in time, although we have lost sight of it for many days, it will appear again, and if it do not die and give rise to suppuration, it will retain its tendency to advance in the same direction in which it had begun to move. The part first protruded is I suppose the head of the animal, and its future progress though very slow and invisible, like the hour hand of a watch, becomes in time very sensible. It seems to wish to escape from its prison, perhaps to assume a new form of existence, but what its next state may be we are at present in perfect ignorance; nor do I know by what means we can trace it farther, for although it be extracted from the body without apparent injury, it soon becomes rigid, after exposure to the air, and no longer gives any evidence of animation, or the power of motion. A future day, I have no doubt, will throw more light on the natural history of this extraordinary animal, which has the power, occasionally, of rendering us very unhappy, of sometimes depriving us of the use of a limb, and even of putting a period to our existence.

---

2. *Experiments on Bile.** The size of the liver, its being found in all animals, the effects of its derangements on general health, and various other considerations, have long induced us to smile at those physiologists who restricted its use in the animal economy to the elimination of some recrementitious matters from the blood, which elimination might be vicariously performed by other organs, as the lungs, without any great detriment to the constitution. The comparatively great size of this organ *in infancy*, when nutrition goes on in a more rapid manner than at any other period of life, and the great emaciation which so generally attends hepatic diseases, led us, at an early period of our professional experience, to conclude that the secretion of bile was intimately connected with chylification or the nutrition of the animal fabric. The experiments of our illustrious countryman, Mr. Brodie, go directly to corroborate, or rather to prove, the truth of the above-mentioned conclusion. These experiments consisted in throwing a ligature around the ductus communis choledochus of an animal, so as to completely prevent the bile from entering the intestine, and then noting the effects produced on the digestion of the food swallowed immediately before or after the operation. These experiments were frequently repeated, and the results were uniform.

It may be proper to state that the operation of tying the choledoch duct in inferior animals, as the cat for instance, is easily accomplished, and at an expense of very trifling suffering; so that any derangement in the functions of the viscera, which follows, cannot reasonably be ascribed to the mere operation. The division of the par-vagum near the cardia of the stomach, and the ligature of the whole extremity of the pancreas, which are operations of much greater difficulty and pain, did not at all interfere with the conversion of food into chyme, or that of the chyme into chyle.

In Mr. Brodie's experiments, which were chiefly on young cats, the production of chyme took place in the stomach equally the same whether the ligature was on the ductus communis or not. But the conversion of chyme into chyle in the intestine was invariably and completely interrupted, when the bile was prevented from mixing with the chyme by the application of the ligature. "Not the smallest trace of chyle was perceptible either in the intestines or in the lacteals." The intestines contained a semi-fluid substance resembling

---

* Mr. Brodie.—Journal of Science, Jan. 1823.

the chyme found in the stomach, with this difference, however, that it became of a thicker consistence in proportion as it was at a greater distance from the stomach, and as it approached the termination of the ileum, the fluid part of it had altogether disappeared, there remaining only a solid substance, differing in appearance from ordinary fæces. The lacteals, under this state of things, contained a transparent fluid, which Mr. Broadie supposed to consist partly of lymph, partly of the more fluid portion of the chyme which had become absorbed.

"I conceive," says Mr. Brodie, "that these experiments are sufficient to prove that the office of the bile is to change the nutritious part of the chyme into chyle, and to separate from it the excrementitious matter." Our author properly observes that, in the very few authenticated cases of total obliteration of the ductus communis in the human subject, there has always been extreme emaciation, shewing that the function of nutrition was not properly performed, so that these cases form no objection to the conclusions which our author has drawn.

In the prosecution of this inquiry, a circumstance occurred which illustrates the great extent of the powers of nature in restoring channels after they have been interrupted or destroyed by ligatures or other foreign injuries. The ligature round the duct was always a single silk thread, the ends of which were cut off close to the knot. If the animal was allowed to live he became jaundiced, as was seen by the eyes and urine. But at the end of seven or eight days, there was an effort made by nature, in several instances, to repair the Injury done by the operation, and to restore the passage of the bile into the intestine. When the animal was killed at the end of the period abovementioned, it was found that, on pressing the gall-bladder, the bile flowed from the ductus communis in a full stream, notwithstanding the previous ligature. On further dissection Mr. Brodie found that a mass of coagulable lymph had been effused, adhering to the ductus communis above and below the ligature, and to the neighbouring parts, inclosing a cavity in which the ligature was contained. The pressure of the latter had caused the duct to ulcerate, without adhesion having taken place of the surfaces which had been brought into contact—and the ligature having been separated, lay loose in the cavity formed by the albumen which had been effused around it. Into this cavity the bile might be made to flow from the upper orifice, and out of it into the lower orifice of the ductus communis—and thus the continuity of the canal intended for the passage of the bile was restored. The physiologist, Mr. Brodie remarks, will not fail to observe the difference between the effects produced by a ligature applied to an excretory tube, and a ligature applied to a sanguiferous vessel. This difference, however, is not invariably observed; for in Dr. Parry's experiments on the carotid of a sheep, it was found that new vessels were sent out from the carotid, traversed round the ligature or obliterated point of the artery, and re-entered the trunk of the same vessel again, thus completely restoring the course of the circulation.

3. *Laryngeal Disease.** Mr. Shaw was called to examine the body of a gentleman, who had died of disease of the throat, the nature of which caused discrepancy of opinion among the medical attendants before death.

The early symptoms had been pain in the throat, with expectoration of blood, followed by difficulty of swallowing, and *copious puriform expectoration.* The physician first in attendance conceived that it was œsophageal stricture, with probable disease of the lungs. A surgeon therefore was employed to introduce bougies, which did no good. A consulting physician considered it disease of the larynx, and that the difficult deglutition might fairly be attributed to spasm of the muscles of the pharynx, produced by the contact of food with the diseased and irritable larynx—the source of the puriform expectoration. Had we never known the sequel of the case, we should have coincided with the opinion of the latter physician; because the symptoms were not those of stricture of the œsophagus, and we have generally, indeed almost invariably, observed difficult deglutition attend ulceration of the larynx.†

Acting on the new view of the case, opium was prescribed, and had an immediate effect in alleviating the dysphagia. The patient, however, gradually sunk, apparently from excessive irritation of the larynx, but with occasional difficulty of swallowing to the last.

*Dissection.* On opening the lid of the coffin, the emaciated living figure was changed to "a large bloated and putrid body," although the patient had been dead but 48 hours, the weather being cool. The tonsils and back part of the tongue were slightly eroded, as was the surface of the epiglottis. The sacculi laryngis were ulcerated, and the edges of the cordæ vocales were thickened and eroded. The lining membrane of the larynx was inflamed and thickened, the marks of inflammation gradually disappearing towards the bronchii. The lungs were very little affected. The upper part of the pharynx was ulcerated, but only in a very slight degree. There was no obstruction in the œsophagus.

Mr. Shaw attempts an explanation of the swelled, putrid, and emphysematous state of the corpse. He thinks the emphysema was produced by a difficulty or obstruction to the exit of the inspired air, "similar to what occasionally occurs in women during labour;" and that the sudden putridity was owing to the presence of this air in the cellular membrane of the body. We must refer to the origi-

---

* Mr. Shaw.—Med. and Phys. Journal, No. 283. Sept. 1822.

† We recently attended, in consultation with Mr. Cosgreave, of Surrey Street, the late Mr. Lewis of Chelsea, who died of ulcerated larynx, or rather we should say, of inanition from the difficulty of getting any thing into the œsophagus, without exciting the most distressing paroxysms of coughing. He was reduced to a mere skeleton before he died, though the *quantity* of expectoration was never profuse, nor in itself adequate to produce the effect described.—*Rev.*

nal paper itself for Mr. Shaw's reasonings and illustrations on the subject. We would just remark, however, that in those cases which fell under our own notice, the difficulty seemed principally to be in *inspiration*, rather than in expiration.

---

4. *Tonic Treatment of Phthisis.** Dr. John Hume has lately laid a paper before the Clydesdale Medical Society, in which we have seen many interesting remarks on that scourge of our country—phthisis pulmonalis. He relates several cases, where, after the first inflammatory symptoms had subsided, gradually increased exercise in the open air, with nutrient, but mild diet, acids, and light tonics, effected a cure, even after purulent expectoration had appeared. He also gives a tolerable exposé of Dr. Stewart's plan, which is not generally known to the profession.

Unless in the first attack of hæmoptysis, when every thing indicated strong inflammatory action, Dr. Hume always found that general blood-letting hastened the fatal termination of the disorder; whereas, local bleeding was often serviceable, if the patient was not previously too much reduced. He has used digitalis with advantage in inflammatory cases, where there was not too much debility—but where purulent expectoration existed, it was worse than useless. Of astringents, he found kino, joined with opium, productive of good effect in lessening expectoration and repressing febrile action. In several cases the superacetate of lead and opium gave temporary relief, and never did any harm. The muriatic acid is also a useful medicine. "An excellent pectoral mixture is made with treacle, honey, vinegar, and laudanum."

"But of all the modes of treating phthisis, either incipient or confirmed, the tonic appears to me to be decidedly the best; and which, though it certainly cannot cure confirmed phthisis, prolongs the patient's life and renders him much less susceptible of those annoyances that unavoidably, for a long time, precede death. But, to be successful, this treatment must be commenced at a very early period. Then, provided there are no violent inflammatory symptoms, frictions, with cold vinegar and water, twice a day, on the breast, or the whole body, till the skin is quite dry, exercise in the open air, or on horseback, in a carriage, on foot, or in a swing, according to circumstances; nourishing, but plain food, taken at such intervals as not to load the stomach, with the occasional use of steel and bitters, are the most proper remedies. Pain in any particular part may be removed by the application of leeches, aromatic embrocations, blisters, or the tartar emetic ointment. From the few trials which I have made with this remedy, I am inclined to have a very favourable opinion of it; for when pustules have been excited by it, they have

---

* Quarterly Journal of Foreign Medicine, &c. No. 17, for Jan. 1822.

never failed to give wonderful relief to the internal symptoms, although they themselves may have occasioned very considerable irritation. In one case, however, where even its frequent application failed to produce pustules, the patient complained of inexpressible anxiety and uneasiness in the epigastric region.

"The greater part of this plan, I confess, is borrowed from Dr. Stewart; although, long before I knew how he treated phthisis, I had appreciated the good effects of bracing remedies, and had employed many of them; and, in debility, or irregular action of the bowels, had often used, with the very best effect, a powder composed of crabs'-eyes, rhubarb, nutmeg, ipecacuanha, and ginger, given so as to prove gently laxative.

"Dr. Stewart was led to the peculiar practice which he has adopted in consumption, from reflecting attentively on some of Dr. Gregory's statements, when lecturing on that disease. In the opinion of that celebrated physician—and, indeed, of that of many others—consumption, in the generality of cases, originates in a scrophulous constitution. What then is the cure, or the attempt at cure, in scrophula? Is it not to invigorate the constitution by a rich and generous diet, by exercise in a pure and open air, particularly near the sea-side, and by the cold bath? If such be the mode of cure directed for scrophulous ulcers on the surface of the body, is it not natural to believe that ulcers of the lungs, probably arising from a scrophulous cause, will be equally benefited by a similar treatment?

"In cases where the skin glows with much heat, where the pulse is firm, strong, and quick, with severe topical pain, Dr. Stewart sees, at once, the propriety of using the antiphlogistic regimen; and in this he entirely agrees with the far greater number of physicians. But, would he do so when the patient is enfeebled, where there is merely chronic inflammation, and where, though the pulse is quick, it is so only from debility? Surely we should not argue, from the patient having a quick pulse, that he should be put on a lowering diet, and use debilitating remedies. Is it not notorious that, on the approach of death, in very old or infirm persons, the pulse beats with a very increased quickness? When the heart has little ability to send forward its contents, its efforts, of course, are more feeble, but, at the same time, they are more frequently made. That quickness of the pulse, then, and febrile heat, which are occasioned by ulceration of the lungs, in conjunction with chronic inflammation, cannot be cured, in the opinion of Dr. Stewart, by such means as will depress the general vigour of the system. If a patient, of an emaciated habit, with feverish symptoms, had foul ulcers on his limbs, would any one of our modern physicians prescribe blood-letting and purging as the best means of curing him? Or would he direct all his attention to the very slight inflammation at the edges of the ulcers, and neglect entirely the constitutional derangement? Would he not try rather, by a nourishing diet, mild cordials and bark, to invigorate the system, that the vessels may recover their native tone, and, instead of a vitiated secretion depending on debility, be enabled to exude a healthy lymph, which may form granulations and consolidate the diseased surface? The very same thing Dr. Stewart wishes to effectuate in cases of internal ulceration and debility. In consumption, he gives nourishing diet, pure or diluted wine, bark, steel, and other tonics; he uses exercise on horseback or in a carriage, or in any other practicable mode; and he employs friction with vinegar and cold water; all with the design of bracing the system, and enabling it to assume that healthy action which alone can render it capable of resisting the sudden vicissitudes of our climate. In short, if he cannot make the weather suit his patient, Dr. Stewart tries to make his patient invulnerable to its attacks. He has been accused of giving wine and beef-steaks to a person who is spitting blood, and at the same time under the influence of strong arterial action. So far from this being the case, he would be amonst the foremost, in such cases, to recommend the antiphlogistic regimen, and even to administer digitalis, a medicine which he unequivocally condemns in phthisis. But even in chronic inflammation, and ulceration of the lungs, he approves of blistering, and makes no opposition to the occasional employment of topical blood-letting. He pays the utmost attention to the regular evacuation of the bowels, and at the same time, takes great care not to give heating cordials."*

When the foregoing observations are viewed in connexion with the investigations of Læennec and Baron, who contend that the softening down of pulmonary tubercles is *not* a process of inflammation, they will not be found devoid of interest. We should bear in mind, however, that the irritation of tubercles very generally causes inflammation in the surrounding parenchymatous structure of the lungs, and that therefore we have to guard against *this*, while we act on the tonic or any other plan calculated to fortify the general constitution and arrest the scrophulous process in the tubercles themselves.†

---

5. *Prolapsus Ani.* In the October number of the Journal Universel, M. Dupuytren has proposed a mode of curing prolapsus ani, which he has put in execution several times with success. He was led to the operation by observing that the excision of piles frequently cured the prolapsus, which often is an attendant on that complaint. The operation consists in cutting off a greater or less number of the projecting folds that protrude beyond the verge of the anus by means of a ligature-forceps, a little flattened at one end, and a pair of scissars, curved on the flat side. He seizes them at an inch and a half from the anus, and cuts them off as much forward as possible in passing towards the rectum. This operation, he observes, contracts the opening of the rectum, somewhat in the same manner as the strings shut a purse, and thus future prolapsus are prevented.

---

* Quarterly Journal, p. 148-9.

† See what we have said at page 801 of the last volume.

6. *Ununited Fracture.** These unpleasant accidents are sometimes, no doubt, the consequence of negligent treatment, in not keeping the limb quiet, and the bones in contact; but we believe they are more frequently the effect of some idiosyncrasy of constitution or mal-habit of body.

*Case.* Mr. C. of Teignmouth, fractured the left humerus near the insertion of the deltoid, in August 1820. Nothing particular occurred till the removal of the splints, when it was found that no union had taken place. He was 30 years of age, and had enjoyed good health, In May 1821, he came under Mr. Earle's care. The broken ends of the bones were quite moveable, and the superior portion was drawn in towards the axilla, and not apparently in close coaptation with the inferior.

" While I was examining him, I was surprized to find that the integuments of the arm and fore-arm, in the space of a few minutes, became covered with urticaria. On remarking this to him, he stated, that he had been subject to this affection for many years; that he never suffered in his health from it; and that the slightest irritation, and even the friction of his clothes, would at any time produce it. This led me to be very particular in my enquiry into the state of his general health, and his various secretions. He had an unhealthy sallow aspect, he perspired profusely, and his perspiration had a peculiar fetid smell, and stained his linen of a brownish tint. His appetite was good, and he slept well; but his bowels were very irritable. Fermented liquors, such as beer and cider, or any irregularity of diet, produced a copious deposit of lithate of ammonia in his urine; although he was not sensible of any dyspeptic feelings, and conceived himself in health. His common beverage was cider. I recommended him to have a seton passed through the arm, between the ends of the bone; which, on a revision of all the circumstances of the case, I was led to hope might be attended with success. I further recommended him to take the opinion of Mr. Brodie, who concurred with me in the propriety of the measure." 192.

The seton was passed, and it was discovered that the fracture had been oblique, with a projecting point occupying the outer part of the inferior portion. Considerable inflammation and a copious discharge of offensive matter followed, with a good deal of fever and constitutional disturbance for some days. Some tumefaction of the soft parts around the fracture took place, and gave hopes of union. No union, however, occurred, although every attention was paid to the general health and to the local coaptation of the bones. The seton was therefore withdrawn at the end of seven weeks. Mr. Green now suggested the application of caustic potash to the ends of the bones. This was done, and some exfoliation took place, while a thickening around the fracture assumed a very firm feel, bearing all the charac-

---

* Mr. Henry Earle.—Med. Chir. Trans. vol. xii.

ter of callus. This, however, became ultimately absorbed, and the limb was once more as weak and useless as ever.

Some other cases are related to shew the probability of a peculiar idiosyncrasy being the principal cause of these non-unions.

7. *Bronchocele.** Mr. Austin has made a comparative trial with the concentrated tincture of iodine, and the burnt sponge lozenges in bronchocele. The patients were two sisters in the same family, and apparently in an equal degree affected. The iodine was more effectual than the sponge, and more speedy in reducing the thyroid tumour.

8. *Enteritis.*† Mr. Wade, Apothecary to the Westminster Dispensary, has published two cases of enteritis, one of which was fatal, and the other was cured. The appearances on dissection are clearly detailed, but exhibit nothing new. We should not have noticed these cases, were it not for the purpose of observing that medical men now-a-days trust too exclusively to sanguineous depletion in enteritis, and neglect certain powerful auxiliaries. If, when they have bled copiously, and as far as the patient's strength will bear, they exhibit the submur. hyd. in combination with opium, they will have the satisfaction of saving many lives. This has long been our practice—and we know it to be the successful practice of one of the first hospitals in this metropolis.

9. *Dilatation of the Female Urethra.*‡ There is a very general prejudice generated in the human mind against the knife, where other means, though more slow and painful, offer a chance of success. It is very praiseworthy in Sir Astley Cooper, whose dexterity with cutting instruments is tolerably well ascertained, to exercise his invention in superseding, on some occasions, the very instruments by which he has gained so much of his celebrity.

The dilatability of the female urethra has been proved by papers in the Transactions under review, from the pen of Mr. Thomas, Mr. Travers, and Sir Astley himself. The latter has here endeavoured to shew a better means of producing this dilatation, than by the introduction of sponge tents, which is liable to the objection of requiring several hours of pain and retention of urine while lying in the meatus. This inconvenience suggested to Sir Astley the

* J. B. Austin, Esq. Med and Phys. Journ. 284. † Ibidem.

‡ Sir Astley Cooper, Mr. Birt, Mr. Chapman. Medico. Chir. Transactions, vol. xii utility of an instrument constructed on the plan of a speculum ani to enlarge the urethra, which would have the advantage of permitting the urine to escape meanwhile. An opportunity was soon afforded in the case of Mrs. M'C. a lady who had, for six months, been labouring under extreme irritability of the bladder, and such pain and difficulty in making water as induced suspicion of stone. On examination, stone was discovered, and Mr. Weiss of the Strand, quickly constructed an instrument which answered the purpose, and an engraving of which is given. On the 7th of January, 1822, Sir Astley, in the presence of Dr. Nuttall and Mr. M'Nab, introduced the dilator at eight o'clock in the morning. At four P. M. Sir A. removed the instrument, and readily felt the stone with his finger, The forceps were introduced and the stone extracted. For a few days after the operation, the patient experienced a severe attack of irritative fever, which was subdued by the usual antiphlogistic means, and our author had the pleasure of seeing his patient gradually restored to health, having never lost the power of retaining her urine. From the facility with which the meatus dilated, Sir Astley thinks there was no absolute necessity for allowing the dilator to remain so long in the passage, and is therefore determined in future to use the dilator for a few minutes only.

*Case* 2. A lady, a patient of Mr. Scott, of Bromley, having been subject to retention of urine, was in the habit of introducing the catheter herself, but broke it one day leaving a portion in the bladder. Sir Astley introduced the dilator for a few minutes and then the forceps, by which he extracted the broken piece of catheter.

The advantages of this operation over lithotomy are very conspicuous. In the first place, it requires but a very moderate knowledge of anatomy. Secondly, it is attended with little danger, unless the dilatation be violently made. Thirdly, the operation may be accomplished with comparatively little pain. If the stone be large Sir Astley thinks the dilatation should be gradual, and repeated for a few minutes or hours every day, till a sufficient extension is accomplished. Fourthly, its greatest advantage is in the preservation of the power of retaining the water, which that by incision does not always do.

To Sir Astley's paper, an Appendix is added, containing communications from Mr. Chapman of Wandsworth, and Mr. Birt of Diss in Norfolk. Mr. Chapman was enabled to extract a female cathether, which slipped into the bladder while a surgeon (not Mr. Chapman) was drawing off some urine, by means of his finger alone. In Mr. Birt's case a calculus was perceived in a female, and by means of two probes the meatus urinarius was so far dilated as to admit a pair of forceps, by which two calculi were successively extracted.

We hope soon to be able to communicate the particulars of a case, where Sir Astley extracted a stone from the male bladder through the urethra, of considerable size, viz. nearly an inch in the longest diameter, and more than half an inch in the shortest. The stone we have seen.

P. S.—This case and others have since been read to the society. A report was spread in some of the surgical circles, that both the instrument and operation were circumstantially detailed by Fabricius Hildanus 200 years ago. The Editor of this Journal, produced to the society the original drawing of Hildanus, and read the text that accompanied it, from which it appeared that no foundation whatever existed for the charge of plagiarism; as Hildanus's instrument was merely for extracting small calculi from the *urethra*, and he never even alludes to the subject of extracting them from the *bladder*.

---

10. *Carcinoma Mammæ.** Mr. Bell, from his official situation, is required to visit regularly a numerous class of female patients suffering from cancer—a disease that does not make a silent and gradual progress, leaving gleams of hope in the tortured bosom till the last; but one that, as Mr. Bell justly observes, is striking and monstrous, filling the mind with horror not to be concealed—the patient dying daily, and conscious that she is dying! yet how seldom does the pressure of pain, or the palpable approach of death elicit from these unfortunate and *practically* philosophic sufferers any expression of impatience or complaint! "They are, on the contrary, calm and placid, if not cheerful, giving an example of unostentatious resignation, and the blessed influence of religion—by witnessing which, the mind naturally reverts to the boasted instances of philosophy in the other sex, which are as nothing in comparison."

Mr. Bell remarks, and no doubt with great truth, that many hopeless cases of local disease, under the name of cancer are sent into the Middlesex wards—some of them tractable, others incurable. An important object is, therefore to alleviate pain, prolong life, and render the disease bearable—to distinguish diseases of a different nature, to know when to use the knife, and when to avoid severe and formidable operations. Mr. Bell proposes, first, to present a history of true carcinoma—then to give the character of other diseases which differ in some circumstances, but are of the same formidable nature—lastly, to return to the consideration of the character and changes of the more common form of the disease, for the purpose of instancing the mistakes to which we are liable in treating it.

1. *Carcinoma Mammæ.* Age has influence on all carcinomatous tumours. They will run their course rapidly in a woman of 45, while they will remain stationary for years, in a woman of 60 or 70 years of age.

---

* On the Varieties of Diseases comprehended under the Name of Carcinoma Mammæ. By Charles Bell, Esq. F. R. S. Ed. Surgeon to the Middlesex Hospital, &c. Med. Chir. Transactions, vol. xii.

" In an old woman, the carcinoma will present one or two small tumours exceedingly hard, and attached to, or incorporated with, the wasted mamma, and with the pectoral muscle. The skin over these tumors will become attached, the surface will become discoloured and livid, and will exhibit a great degree of venous vascularity.

" These hard lumps will remain very long stationary, having a rosy surface slightly ulcerated. The ulceration will indeed sometimes close, and the tumor remain inactive, while a constitutional disease is making slow progress. The patient, excessively extenuated, will at last sink, from the continuance of a peculiar hectic, attended with pains in the lower part of the spine, hips, and shoulders." 216.

2. The true carcinoma belongs to the turn of life. The mamma sympathises with the condition of the uterus—pains strike through it—it swells—the turgescence and tension subsiding, a more partial hardness is left, with irregular margins mixing with the substance of the breast, and progressively extending till the whole gland is involved in a tuberculated or knotty condition. Large blue veins become apparent on the surface ; and in the mean time the patient's strength and flesh decline.

The nipple is now drawn in, and incapable of erection, and ultimately the skin is puckered in, the whole adhering firmly to the mass below. Hæmorrhage from the nipple is an occasional occurrence and an unfavourable symptom. The axillary glands are early affected in such cases.

" A true carcinoma may begin very differently, and exhibit a different exterior character. A small hard tumor is felt deep seated in the mamma. It is difficult to distinguish whether or not it is a part of the proper gland. Its progress is generally this ; it becomes painful, it draws nearer to the surface, it becomes attached to the mamma and to the skin, and is gradually incorporated with them. The skin becomes discoloured, the surface becomes moist, and the patient is alarmed lest a sore should break out.

" It does ulcerate, and begins to discharge. The bottom of the sore is foul and sloughy, the smell is offensive, and the constitution sympathises with the state of the sore.

" The whole gland is now hard, and adhering to the pectoral muscle. The edges of the sore are particularly hard, of a dark red, and have a glazed appearance. The edges have not risen with everted and curling margins : they are rather depressed under the general convexity of the tumor, and, as it were, condensed into a smaller volume. This will certainly be the appearance presented in a fat woman." 218.

In proportion to the depth of the ulcer the surrounding hardness extends, and, when grasped, it feels like stone.

3. The disease commencing in the mamma, will sometimes propagate itself by building up its peculiar structure in the cutaneous tissue, rather than in the glandular. Tubercles will be felt in the skin around the nipple, extending to the breast, neck, and shoulders. These tubercles become painful, and are soon attached to the skin. They first assume a high red colour, then a yellowish transparency in the centre, ultimately opening into a corroding and wasting ulceration.

4. Another form of the disease is when the breast presents a tumour elevated, tuberculated, and remarkably firm, without elasticity, and fixed to the side. The surface is granular, and of a deep or dark red colour, with a bluish cast. This tumour ulcerates and sloughs—bleeds profusely—is propagated by tubercles under the skin towards the sternum and clavicles. Effusion into the chest is an early accompaniment of this form of disease.

5. *Impostumated Cancer.* A deceptious appearance sometimes takes place in the progress of cancer.

The mamma not yet ulcerated, will swell, soften, point, and finally burst, with all the appearance of a scrofulous abscess, thus affording a hope that the disease is not of a malignant nature. But a large sloughy and foul chasm is presently disclosed; the surrounding parts become harder and tuberculated—the edges get everted—and real cancer is established.

6. "It is important," says Mr. Bell, "to observe the variations in the aspect of cancer. The local disease makes its progress in two ways: 1. by *ulceration*, that is by absorption; 2. by *mortification*, and sloughing of successive portions. And accordingly the aspect of the sore varies in a very remarkable manner. To-day, the symptoms shall be mild, the matter bland, and the breast altogether in a tranquil state: to-morrow, it will be full of pain and tumid, the discharge thin and corrosive, certainly acrid, the edges of the sore sharp and of a fiery redness, and the bottom full of sloughs.

"But this will not continue; the excited action will again subside; once more the ulcer appears clean, and shows a disposition to heal, and even to cicatrize. Sometimes a cancer will close, and be quite healed; but the skin, though closed, is not sound, it is too vascular and of a dark lake colour, and the parts beneath are hard and irregular." 222.

7. *Constitutional Condition.* This is melancholy enough. Great bodily suffering and mental misery—hectic fever—emaciation—countenance pale and anxious, with a slight leaden hue—features sharp—lips and nostrils slightly livid—pulse frequent—pains lancinating, and in the exposed surface burning sore—pains, like rheumatism, extending over the body, especially to the back and lower parts of the spine, then the axilla—swelling and immobility of the corresponding arm—in process of time, nausea and indigestion—tickling cough—severe stitches in the side—pulse becoming rapid and fluttering—breathing anxious—these are the phenomena that attend the victim of cancer in its fatal career to death.

8. Mr. Bell describes an hydatid carcinoma mammæ which, when the disease assumes its full volume, presents a very remarkable appearance.

"The tumor stands prominent; its base, that is its connexion with the side, is not the place of its greater diameter. It measures less round the base than a little removed from the side. It is not hemispherical, but of a squared or angular form. The nipple is not drawn in, but stands prominent, and with a natural appearance. The veins upon the surface are of a great size, and yet perhaps only bear a relative magnitude to the tumor. The tumor to a very late stage is loose from the pectoral muscle. It is irregular, and not squally soft or elastic; the elasticity, at certain points, is so considerable as to distinguish it from the common carcinoma.

"In operating on such a tumor, it is much easier to turn it off from its connexions than to dissect out a common carcinoma. It is, however, a bloody operation, for many branches of arteries require to be tied.

"This tumor when cut into, does not exhibit a concentrated mass, but is distinguishable into parts or clusters of lesser tumors. When these subdivisions are cut into, they present the most common carcinomatous appearance, being firm in texture, and having the ligamentous bands both forming areolæ and diverging lines, and these are distinguishable by their whiteness from the matter they embrace. In the interstices of the tubercles, some larger bags or cells are found, of a yellowish or amber colour. These cells are of various sizes, and the larger ones contain a dark fluid like blood, or bile." 225.

This species of carcinoma makes its attack at the climacteric period —is slower in its growth—and the mass may be extirpated with more sanguine hope of permanent advantage.

9. *Acute Carcinoma Mammæ.* This form is more rapid in its course, and severe in its symptoms than any of those before described.

"It begins in a hard tumor or kernel deep-seated in the mamma. At first it is loose, and slips about under the finger. After a month or two it becomes attached to the skin and the surface is discoloured. The hardness extends to the whole breast, but some one part projects and is more discoloured, exhibiting a purplish red colour. The surface is shining and elastic, as if it contained a fluid. During this stage the pain is severe, and the tumor shoots like a whitlow.

"The breast now enlarges, not uniformly, but in successive divisions of the tumor; and as it extends, the structure of the skin is evidently affected, and partakes of the disease. The glands of the skin appear to be enlarged, and the surface becomes studded with small white spots, and as the dark red swelling increases, these spots become more distinct. On the most projecting division an oozing takes place; it promises to suppurate, but no proper suppuration is established. Suddenly the whole tumor swells and inflames with more threatening redness, and increase of pain. Now the countenance indicates pain and anxiety. The skin has a suffused yellowish hue, and the patient feels great lassitude and prostration.

"At this stage the disease is hardly to be distinguished from the fungous tumor to be next described; but when it opens and displays its last horrid aspect, it is sufficiently distinct. After much pain, the enlarged tubercles of the skin become black on the points and break, and discharge a little blood; and after this, serum drains through the dressings. Suddenly the surface becomes extensively sloughy, and the breast is deeply excavated." 227.

10. *Acute Fungous Tumour of the Mammæ.* It is necessary to be acquainted with this form, in order not to give false prognosis of the disease. The patient, instead of lingering out four, five, or six years, may be hurried to the grave by this species in as many weeks. The tumour occupies the whole gland—the base is hard, and firmly attached to the pectoral muscle—the tumour and its inflammation are characterized by a dark purple or lake colour—it seems about to point in three or four places—the surface then becomes intensely red—and of a granular appearance. When it opens, instead of a fluid, a soft fibrous substance is disclosed, which bleeds—the pain is throbbing and burning—the patient becomes weak, hectic, and yellow—and she sinks rapidly, probably after losing a quantity of blood from the accidental disturbance of the fibrous projectory substance.

11. *Structure of Carcinoma Mammæ.* On cutting through the true carcinoma, we are struck with the density of its composition. A hard centre is seen, with firm white bands diverging from it, and communicating so as to form meshes, between which a distinct substance, a new matter is found. Although the fibrous bands and the intervening soft matter are new textures, it is difficult to say which has the priority in point of formation. Mr. Bell is inclined to think the firm bands are first formed, and that the softer substance is afterwards secreted and deposited within the embrace of the meshes.

"As it is the constitution of many of the natural organs to remain as it were suspended in their growth, and at a certain period to assume activity, and fully develope themselves; so, unfortunately, cancerous tumors have the laws of their constitution; and, after a period of deceitful indolence, they acquire activity, and the structure, which was before too dense for inspection, is fully blown and developed." 231.

A question of great practical importance naturally arises here:—is there any appearance which, during life, will indicate how far these radiating bands or filaments may have shot, in order to determine the propriety of an operation? It is but too certain, Mr. Bell observes, that they may have shot far beyond the irregular hardness of the tumour, as felt externally. "But the retraction of the nipple will indicate the general extent of these bands."

"It is of much importance to ascertain the cause of the retraction of the nipple. It is produced by the peculiar carcinomatous texture of filaments, which rise up from the original centre of the disease, and extend betwixt the ducts of the nipple; these, by condensing and destroying the spongy texture of the part, cause its retraction. Now, it unfortunately happens, that by the time these ligamentous bands have, by extending in one direction, produced the retraction of the nipple, they have, in another, extended into the cellular membrane, beyond the margin of the gland." 233.

We daily see, he says, the operation of extirpation determined on, *because the nipple is retracted, and because true cancer is thereby announced.* Yet, to him it is quite clear, that if the nipple be fully retracted for any considerable time, the operation has been too long deferred.

We have thus given a very full account of Mr. Bell's paper, because the subject is important, the author's field of observation is extensive, and his powers of discrimination are unquestionable.

---

11. *Diabetes Mellitus.** Dr. Heineken, of Madeira, has published a case of diabetes mellitus in our respected cotemporary, where the patient had fallen away from being robust to a state of great emaciation. He was passing eight or ten quarts of urine daily of a saccharine quality, and had the usual symptoms of diabetes. The following pills were ordered:—pulv. scammon. ℈j. pulv. opii, ℈j. hydr. submur. gr. v. ant. tart. gr. ij. To be made into twelve pills, one to be taken three times a day. A warm bath every night—the chest, arms, and abdomen to be rubbed well with sweet oil every morning. The food to be entirely animal. This treatment, with little variation, was continued from the 3d of June to the 17th July, when it was noted that the patient perspires freely—that the urine is healthy—that the quantity is about three quarts daily. A nearly similar mode of treatment, however, was continued till the 8th of October, when he was considered as cured. The quantity of solid opium taken during the treatment was about six hundred grains; and during eight days of that time, he took fifteen grains daily.

---

12. *Tetanus cured by Ol. Tereb.*† Mr. Hutchinson has stated a case, in a much respected cotemporary, of tetanus occurring without any evident cause, in a man long afflicted with epileptic attacks. He was a prisoner in the Nottinghamshire House of Correction. Mr. H. finding, on his visit one morning, that the patient presented most of the usual phenomena of tetanus, he abstracted 30 ounces of blood, and exhibited fifteen grains of calomel and two grains of opium. Next was administered a brisk purgative enema, containing one

---

* Dr. Heineken.—Med. Repos. No. 112.

† B. Hutchinson, Esq.—Med. and Phys. Journal, No. 288.

ounce of the oleum terebinthinæ, while a large blister was applied to the back. The enema taking no effect, half an ounce of the turpentine was ordered by the mouth every two hours. Next morning our author was agreeably surprized to find that the patient could open his mouth, and that the whole of the tetanic symptoms were dispersed. He had taken, in all, two ounces of the turpentine, which freely evacuated his bowels. We would hint our suspicion that the case was not one of real tetanus, from the great facility with which the symptoms gave way. Howpver this may be, we have nothing to object to the mode of treatment employed.

---

13. *Oxalic Acid.** A paper evincing much discrimination, zeal, and patient labour, has lately been read before the Medico-Chirurgical Society of Edinburgh, and published in our highly respected cotemporary, for April, of the present year. We can only present our readers with an abstract of the more interesting and prominent features of this experimental essay.

It is well known that oxalic acid has long been used, *in small quantities*, as an ingredient in lemonade, and without any deleterious effects. Its poisonous qualities appear to have been first pointed out by the late Mr. Royston, in 1814. In the succeeding year, Mr. Thomson made some interesting experiments on this acid, and seems to have been the first to ascertain one of the best antidotes—chalk.

The authors of the present paper made many experiments on animals, and enter fully into the question respecting the modus operandi of the poison on the stomach and general system. The following conclusions were drawn from these experiments.

"1. Concentrated oxalic acid renders the mucous epidermis brittle and less adherent. It dissolves the other coats; but during life, this action never extends beyond the surface of the corion, and seldom so far; hence its action on the living system is more like that of the pure irritants, producing extravasation of blood within the tissues, and into the cavity of the stomach, and little chemical decomposition.

"2. But its action on the dead stomach is so rapid, that, if the examination of the body be delayed a few minutes, the whole corion, and even the other coats, will be found dissolved; and the diluted acid will also have the same effect, though more slowly. Hence it is easy to explain why Mr. Thomson, in his experiments, found so much apparent corrosion. In fact, he always allowed an interval to elapse sufficient for the acid to act extensively on the dead tissues.

"3. The chemical action of oxalic acid is not owing, as Mr. Thomson conjectures, to mutual decomposition of the acid and sto-

---

* Drs. Christison and Coindet.—Edinb. Journal, April 1823.

mach: it is one of pure solution, in which the acid and the animal principles of the tissues remain unaltered." 170.

Another series of experiments were made to ascertain whether the deleterious properties of the oxalic acid were impaired or increased by dilution—and it appeared that dilution rendered the poison infinitely more speedy in producing death than when in a concentrated state—and this, in general, without leaving any cognizable trace of organic change in the textures of the stomach to which the acid was applied.

The next subject of enquiry was whether these effects could be attributed to a sympathetic impression conveyed by the nerves from the stomach to distant organs—or by absorption. Our authors come to the conclusion, that when the stomach is organically deranged by the poison, the brain, heart, and other parts, are *sympathetically* affected, but that when the impression on the stomach is unattended with any cognizable trace of the application of the poison, then the other organs are affected by *absorption.* Neither the experiments nor the arguments appear at all convincing to us—and this hair-breadth distinction seems to be a refinement of a very equivocal character. At the same time we do not doubt the fact of absorption occasionally taking place, since death is produced by the introduction of the poison into various other textures of the body, as the peritoneum, pleura, cellular tissue, &c.

Our authors made numerous experiments with the view of detecting the oxalic acid in the blood and other circulating fluids—but "they never could detect the slightest trace of oxalic acid even in the vessels nearest the seat of absorption." They also examined the chyle in the thoracic duct, after the poison had been introduced into the intestines, but with no better success. On this point, therefore, they are at direct variance with Mr. Thomson and Dr. Perey, who affirm, that when the poison was introduced into the stomach or rectum, the blood in the meseraic veins, in the lungs, and in the heart, frequently reddened litmus paper. Considering how many other unequivocal proofs of absorption are given by our authors, it certainly seems not a little surprising, as they themselves acknowledge, that no atom of the absorbed substance could be detected in the circulating fluids. They think the decomposition, and consequently disappearance of the poison, most probably takes place in the lungs.

Another subject of investigation is the inquiry on what organs is the power of the poison exerted from sympathy or absorption. The symptoms and dissections led our authors to the conclusion that these were the spine, the brain, the heart, and the lungs. The order of the phenomena indicated that the primary action of the poison was on the spine and brain—and that the heart and lungs were affected secondarily through injury done to the nervous system.

Our authors next proceed to a history of the poison, as far as relates to the human subject. This history is very defective, for there are not above a dozen of cases recorded in medical works—and but few of them accurately and circumstantially detailed. The following, however, is a summary of our knowledge of the symptoms and post mortem appearances.

Of all poisons (excepting perhaps the prussic acid) the oxalic acid is the most rapidly and unerringly fatal. The first symptoms are burning pain in the stomach—vomiting, commonly of dark matters, and without relief. Death generally happens too soon for the occurrence of gastritis—indeed, inflammation of the stomach does not seem to be a natural consequence of the introduction of oxalic acid. The bowels are seldom affected. The vascular system suffers great depression. The pulse, in almost every case, became imperceptible, accompanied by deadly coldness, clammy sweats, and sometimes livor of nails and fingers. Most of the patients, whose cases are recorded, experienced great disorder of the nervous system, and convulsions usually terminate the scene.

In respect to post mortem appearances, the accounts are very contradictory, but they may probably be reconciled by attention to the *time* intervening between death and dissection. The brain has rarely been examined in these cases, and we find no accounts of the state of the lungs, heart, or blood. The stomach has generally been found to contain a quantity of fluid, of a thick and viscid, or dark and coffee-ground appearance, probably consisting of extravasated blood altered by the poison. The state of the membranes of the stomach is very different, according as the poison has been concentrated or diluted; and according as the examination has been soon or late after death.

In respect to the treatment, little that is satisfactory has been obtained, since, unfortunately, the effects of the poison are generally beyond the control of art before medical aid can be procured. "Yet neither the chemical action nor the absorption of the poison is so rapid, as not to be obviated by the prompt employment of certain remedies." The general rule in toxicology—that of attempting to dislodge the poison from the stomach, is here inconvenient or dangerous; for the time lost in administering emetics would be fatal to the patient. Even the mode lately recommended of extracting the poison from the stomach by means of a syringe would be too tedious. The substances proposed, therefore, as antidotes, are alkalis, chalk, and magnesia. The first are inadmissible, not only as being too acrid and corrosive for the gullet and stomach, but also as being poisons themselves when combined with the oxalic acid, in the form of alkaline oxalates. In respect to chalk, Mr. Thomson found that the oxalate of lime produced no inconvenience when exhibited to animals—and that chalk itself, given after the dangerous symptoms had begun, speedily removed them, and restored the animals to health. Magnesia, however, whose oxalate is equally as insoluble in the stomach as the oxalate of lime, is preferable to all other antidotes, as it is free from the inconvenience of setting loose a great quantity of fixed air, which is extricated when chalk is used. Nevertheless chalk should be exhibited whenever it can be procured quicker than magnesia.

As to the after-treatment little need be said, as the practitioner must act according to the symptoms. As the oxalic acid occasions a remarkable depression of the nervous and vascular energy, it is probable that stimulants will generally be proper, especially as inflammation does not seem to be a common sequel of the application of the poison to the stomach.

With respect to medico-legal researches, when oxalic acid has been swallowed, we can only make room for the following extract, touching the process to be employed for analysing the suspected matter.

"The stomach is to be washed with pure water, and if disorganized, preserved for analysis. The washings, the contents of the stomach, the vomited matter, and the disorganized tissues and suspected articles of food, are to be boiled separately, a little pure water being added if necessary. If chalk or magnesia has been used as an antidote, what remains on the filter (except that from the tissues) is to be preserved for analysis. The filtered fluid is to be tried first with litmus paper, and then by the three following tests—the hydrochlorate of lime, the sulphate of copper, and the nitrate of silver.

"1. Decolorize the fluid, if necessary, with chlorine. The hydrochlorate of lime, dropped into a solution containing oxalic acid, or an oxalate, especially the latter, throws down an insoluble oxalate of lime. But it also precipitates with the carbonates, sulphates, phosphates, tartrates, citrates, and with all their acids but the carbonic. The following mode of procedure will serve to distinguish it from these substances. The nitric acid will not take up the sulphate of lime, but a few drops of it dissolve the oxalate. The hydrochlorate acid will not dissolve the oxalate, unless added in very large quantity, while two or three drops will take up the carbonate, phosphate, tartrate, or citrate.

"2. Decolorize a second portion of the fluid with chlorine. The sulphate of copper precipitates oxalic acid bluish-white, and the oxalates pale blue. This is a test sufficiently delicate, especially if any free oxalic acid is previously neutralized with potass; and it is also a very useful one, since the sulphate of copper does not affect fluids that contain sulphuric hydrochloric, nitric, tartaric, citric acids, or their ordinary salts. But it precipitates the carbonates, and throws down phosphoric acid, whether free or combined. The oxalate, however, is easily distinguished; for it is insoluble in hydrochloric acid, while a few drops of that acid at once take up the phosphate or carbonate.

"3. The nitrate of silver produces a heavy white precipitate with oxalic acid, and still better with the oxalates; and this precipitate, when dried and heated over a candle, becomes brown on the edge, then of a sudden fulminates faintly, and is all dispersed in white fumes. When impure, it deflagrates like gunpowder, and when in too small quantity to be collected, the filtering paper burns, as if steeped in nitrate of potash. This is a very characteristic and delicate test. From a quarter of a grain of oxalic acid dissolved in 4000 parts of water, we have procured enough of the powder to show its fulmination twice. The precipitation alone cannot be trusted to; for it may equally take place with hydrochloric, phosphoric, citric, or tartaric acid, and likewise with the alkalis. But when the test of fulmination is tried, there is no chance of its being confounded with any of these, except perhaps with the tartaric and citric acid. The compounds of these acids with silver, we find, possess properties, that will render the nitrate of silver one of the simplest and most correct tests for distinguishing them from each other, and from oxalic acid. The nitrate of silver becomes brown under exposure to heat, froths up, then deflagrates slightly, with the discharge of white fumes, and a large quantity of dull, ash-grey, crumbling matter remains, of a very peculiar fibrous structure. The tartrate of silver becomes brown, and froths up like the citrate, white fumes are discharged without even deflagration, and there is left an ash-coloured botryoidal mass, encrusted outwardly with silver.

"If magnesia or chalk has been given as an antidote during the patient's life, the oxalate of magnesia or lime may be mingled, in the form of powder, with the contents of the stomach, or with the vomited matter. The powdery matter is then to be separated by elutriation from what remains upon the filter during the previous process. If magnesia has been the antidote employed, it is only requisite to boil the powder in pure water for a few minutes, and then subject the filtered fluid to the three tests described above. For the oxalate of that earth is sufficiently soluble to furnish, even with a single ounce of water, a solution in which all the foregoing characters may be observed. If the antidote employed has been chalk, then the powder is to be boiled for 15 minutes, with half its weight of pure subcarbonate of potass, dissolved in 20 or 30 parts of water. A mutual interchange then takes place, and the solution contains oxalate and carbonate of potass. In applying the tests to this solution, the free alkali is to be previously neutralized with hydrochloric acid, when hydrochlorate of lime or sulphate of copper is to be used, and with nitric acid, before using the nitrate of silver. In the last case, there ought to be as little excess of acid as possible, because the oxalate of silver is soluble in nitric acid." 198.

---

14. *Vaccine Disease and Measles.** We apprehend that the Hunterian dogma respecting the incompatibility of two processes or constitutional diseases at the same time, is now nearly obsolete—at least that the rule is far from being absolute and without exceptions. We have very lately seen two cases of chicken pox (or it may have been modified small-pox, for the children had been vaccinated) co-existing with well marked measles. We are ready to admit, however, that it is sometimes exceedingly difficult to distinguish between eruptive diseases. Measles, and even scarlatina, will sometimes throw out a miliary, nay, a varioloid or pustular eruption, very puzzling and perplexing to the practitioner. The case before us was one of a

* Mr. S. Gilder.—Med. Chir. Transactions, vol. xii.

child vaccinated in a house where the brothers had measles. On the third day from vaccination the child itself exhibited the morbillous eruption. On the fourth day the punctured arms shewed that vaccination had taken—and, in short, both diseases went through the regular stages quietly and amicably together.

---

15. *Ascarides.** Dr. Howison, of Edinburgh, proposes a manual or surgical operation for the removal of ascarides. The patient is to place himself in a semi-bent position by leaning forwards, and then to introduce the middle finger of his right hand, well smeared with tallow grease or hog's lard, up as high as he can into the rectum, and then scoop out, as it were, the infesting vermin from the inner surface of the gut. This operation is to be repeated every evening till the enemy is completely dislodged.

---

16. *Petechiæ Hæmorrhagicæ.*† An interesting case of this disease lately happened in the practice of Mr. Pretty, a very intelligent surgeon, of this metropolis. The patient was a little girl, nine years of age, who had spent six years of that time in the West Indies, whence she lately returned. On the 1st of February, 1823, she appeared a little unwell, and on the 2d, was attacked with sickness, severe pain in the epigastric and umbilical regions, some thirst, and fever—tongue furred, pulse accelerated, respiration quickened, with head-ache, and vomiting of every thing taken. Some calomel, colocynth, and extract of poppy ordered to open the bowels. 3d. Bowels not opened, but the gastric irritability much lessened. A cathartic mixture procured several fetid stools. At mid-day Mr. Pretty was summoned to the little patient, who had still pains in the head and stomach, suffused face—tunica conjunctiva injected—insides of the hands of a dark red colour, breathing frequent, with a troublesome cough, and increase of fever. Bled to six ounces. Some nitre and antimonial powder. At this time petechiæ, of the size of pin's heads, were observed about the arms, breast, abdomen, and right leg. Upon the other leg was a large blue spot under the cuticle, of the size of a six-pence. Some sanguineous discharge from the labia pudendi. 4th. Had a restless night, the fever having increased; function of the lungs much impeded, threatening suffocation—great determination to the head—pulse very rapid and hard, petechiæ increased—the purple spot on the leg enlarged. Mr. Bagster (Mr. Pretty's partner) saw the patient to-day, and they agreed on another bleeding as the most likely means of preserving the head and lungs from serious injury. Ten or twelve ounces were taken from the arm, which produced syncope, and an apparent amelioration of all

---

* Edinburgh Journal, April 1823.

† Mr. Pretty—Med. and Phys. Journal, No. 290.

the symptoms. In a few hours afterwards, however, the fever again got up; and Dr. James Johnson saw the patient. He recommended the exhibition of the mineral acids. In the evening she appeared sinking, and expired at eight o'clock the next morning. The blood first drawn exhibited no serum for a space of eight hours; but after 20 hours a small quantity of serum was apparent. One cup of the second bleeding showed a coat of coagulable lymph nearly half an inch thick, like very soft jelly, with very loose crassamentum, so tender as to be easily broken down with a spoon into a soft pulpy mass. In this cup there was little or no serum. The other portions of blood, which were not taken, however, in a continuous stream, shewed no buffy coat, and very little serum, seemingly as if the blood had become so altered as to be incapable of separating into its constituent parts.

*Post-mortem Examination.* The eye-lids, in addition to the parts abovementioned, thickly studded with petechiæ, and many of a large size, appeared upon the back of the trunk. The brain showed merely some increase of vascularity. In the thorax there were adhesions in the left side, but not of recent formation. The lungs seemed loaded with blood and mucus, but their parenchymatous structure appeared sound. About half an ounce of water in the pericardium —the external surface of the heart of a pale hue, and about twenty small petechiæ on various parts of it, more particularly at the junction of the auricles and ventricles. In the abdomen, all appeared, at first, natural, excepting the stomach, which was distended with air, and thickly spotted with petechiæ, plainly seen through its peritoneal coat. On opening this viscus the petechiæ were still more distinct, about the size of split peas generally. No petechiæ were visible in or on the intestines. Dr. Gregory and Mr. Bagster were present at the examination.

Dissections of petechiæ hæmorrhagicæ are not very numerous, and the above case is very interesting as showing decidedly that the petechiæ are not confined to the skin, but liable to take place in the most vital organs, as the heart and stomach. The treatment of this curious disease is still involved in considerable obscurity, and is far from being settled. The blood of this little patient had a most remarkable and indescribable appearance.

---

17. *Tumour in the Neck.** All operations about the neck are accompanied with more or less danger, and are therefore deserving of record. In the present instance, a boy of six years of age was admitted into the hospital, having a tumour occupying the whole of

* Case of a large glandular tumour in the neck removed by J. P. Vincent, Esq. Surgeon to Bartholomew's Hospital.—Med. Chir. Trans. vol. xii.

the right side of the neck, extending from the ear to the clavicle, and laterally from the edge of the trapezius to the trachea, projecting in a proportionate degree beyond the natural contour of the neck. It had a lobulated feel, the integuments being free, and the mass appearing, at first view, moveable, but on being firmly grasped, it was evidently fixed at its basis. It was not painful, and the boy was in good health. The tumour was removed on the 10th of November, and the steps of the operation we shall give in the words of the author.

" Two semilunar incisions were made from the ear to the clavicle, including a portion of skin between them. The integuments and the mastoid muscle, which extended over the anterior portion, were dissected off. The tumour, which was divided into many portions, was drawn out, and a large mass was removed without much difficulty, by cutting through the firm membranous septa, which were interposed between the lobes. There still, however, remained a considerable portion in connexion with the large blood-vessels, extending from behind the clavicle to the mastoid process. The whole of this was removed, care being taken to draw out each portion from its situation, and to divide the septa with the edge of the knife turned towards the tumor. One portion of the tumor passed behind the internal jugular vein, and another was in contact with the pleura. During the operation there was very little hæmorrhage, and it was only necessary to tie one small artery. The situation and magnitude of the tumor rendered it impossible to avoid wounding nerves of importance. The nervus accessorius, and some branches of the cervical plexus were divided." 248.

For some time the wound did not go on well. Erysipelas supervened on the third day, extending over the face. On the 10th there was difficulty of breathing, with a very disordered state of the bowels, which yielded to purging and blisters. The wound healed, and the child was discharged from the hospital in about five weeks after the operation, there being still some difficulty of breathing, cough, and night perspirations. He soon after fell a victim to measles.

---

18. *Acetate of Morphia.** " Having lately seen much benefit derived in substituting the acetate of morphia for the tinctura opii of the London Pharmacopœia, and as I think it would prove of considerable utility, if generally adopted, I am induced to state the following mode of preparation, which, from several experiments I have lately made, I find to be the most economical, and to answer sufficiently every purpose of a more expensive process.

" Five ounces of good dry opium, broken small, are to be put into a covered vessel with from one quart to three pints of water,

* Fred. Joyce.—Med. Repos. No. 113.

and gently boiled until the whole of the soluble matter appears to be taken up. Pass it while still hot through a fine strainer, and, having poured a small quantity of boiling water on the residue, and having strained and mixed the liquors, add so much calcined magnesia that, when boiled for a few minutes, the supernatant liquor shall have entirely lost its bitter taste: the whole is then to be poured on a filter, and the magnesia precipitate collected and dried, which having been put into a retort, pour upon it one quart of alcohol, and apply a gentle heat to bring it to ebullition, at which state it may be of advantage to keep it for a few minutes, in order to entirely dissolve every portion of the morphia. The solution is now to be passed, as quickly as possible, through a filter, the residue is to be washed with a little hot alcohol, and the filtered liquors are to be mixed; return them into a clean retort, and draw off about one half of the spirit. The contents of the retort are now suffered to cool, when the greater part of the morphia (perfectly pure, with the exception of colouring matter) will be found crystallized on the bottom and sides of the glass. Acetic acid, to saturation, being now added, the process is to be finished with a sufficient quantity of distilled water to make exactly one quart.

"It will be seen, by turning to the Pharmacopœia, that, in this formula, the quantity of opium is doubled. It is, however, of no consequence, as the medical practitioner can, at any time, add either distilled water or proof spirit, and by that means bring it to the strength ordered by the College. In either of these states it will be found to possess every advantage that is derived from opium, without producing the disagreeable headache generally attending the use of the common laudanum.

"As this method may be objected to as not making a perfectly pure acetate, the colouring matter may be removed by recrystallizing the morphia previous to its solution in acetic acid, or afterwards by the use of well-burnt animal charcoal, previously digested in muriatic acid: but as the loss in strength by either of these methods is often considerable, and colour in medicine being of very little moment, this additional purification would, in general, be found an evil. It is also, perhaps, necessary to observe, that whenever a solution of morphia in alcohol is to be filtered, it should be kept warm; otherwise the salt deposits on the filter, and the product is lost. The method I find to answer best, is to have a double copper funnel connected at the pipe, and the space between the two filled with boiling water. In this manner the whole of the fluid in the inner funnel is kept hot until it has passed through the filter; it is also furnished with a cover, which fits air-tight, in the middle of which is inserted a long-necked funnel, which reaches nearly to the bottom of the filter, and by which means it is refilled without removing the cover. This little apparatus is extremely useful for solutions in ether, the outer space being in this case filled with a freezing mixture."

19. *Tumour in the Vena Cava,** J. C. a native of the West Indies, aged 26 years, was admitted into the Pennsylvania Hospital on the 13th August, 1819, with a scirrhous testicle combined with hydrocele, for which he underwent operations. An enlargement then took place in the direction of the spermatic chord, and next the abdomen swelled and reached a great size, with lancinating pains, and constipation, from pressure on the intestinal tube. A mercurial course gave remarkable but only temporary relief. On the 7th of April, 1820, he died, "having all the appearances of internal scirrhus."

"On opening the body, an enormous tumour was found adhering to the peritoneum on the right side, and enveloping to at least one half their width, and with the firmest adhesions, the cœcum and parts of the colon and ileum, on the same side. A process of the same mass extended into the pelvis, included the rectum in the same manner, and adhered intimately to the urinary bladder. Another lobe of the tumour lay higher and deeper, and included the right kidney so completely that it could not, at first, be seen. A large fleshy process extended upwards, separated the duodenum several inches from the spine, adhered to the vena cava, embraced the whole circumference of the aorta, and finally became continuous with the diseased substance which formed nearly the whole of what appeared to be the liver. At the posterior part of the tumour was found the enlarged spermatic chord, extending to the place at which it had been tied, during life, and forming a blunt projection there, which had been felt externally. Its composition was similar to that of the rest of the tumour. The whole presented the mixture of cartilaginous bands with cells of softer matter, which occurs in scirrhus; occasionally with coagula of blood, and in the liver, with several small abscesses. The right kidney was very much enlarged from distention of the pelvis and infundibula which were filled with urine. The internal and tubular portion of this viscus was nearly removed. The ureter was of the usual size, but the passage of fluids through it was rendered difficult by sudden and acute turns. The other kidney was healthy. Much bloody fluid was found in the abdomen and the left side of the thorax. There is no memorandum of the right side." *336*.

The vena cava inferior was distended to an enormous size, by a kind of polypous tumour, four and a half inches in length, and nine-tenths of an inch in thickness, tapering gradually to both ends; but adherent in some places to the parietes of the vessel.

We consider the phenomenon which has given title to this paper in our respected cotemporary of the West, as quite a subordinate one in the series of morbid structures, and very doubtful as to its ante-mortem or post-mortem formation.

* Dr. Coates.—Dr. Chapman's Philadelphia Journal, No. 8.

20. *Bilious Remittent Fever.** We have always endeavoured to impress it on the minds of our junior brethren, that fevers are greatly modified by the local nature of the remote causes, the climate, and the constitutions of those attacked. Some writers would persuade us to a very simple pathology and a very uniform mode of treatment in fevers of all kinds; as if their seat was always in a particular structure of the body, and the nature of the lesion constantly the same. And even where the post mortem appearances are nearly the same, the fevers, of which these are merely effects or consequences, are often very dissimilar, and require different modes of treatment.

Mr. Goodisson, the author of the interesting little work before us, has been serving for seven years past in the Ionian Isles, and has given the British public an entertaining account of many places famed in Grecian song and history, and now enjoying peace and prosperity under the English flag. He has added an appendix on the bilious remittent fever of the country, of which we shall take some notice in this place. In his remarks, he begs to be understood "as not attempting to decry the use of blood-letting in fever in general—a remedy which, for six months of the year, he always found to be the sheet-anchor in the treatment of the Mediterranean fevers, and the utility of which the late Dr. Irvine (and he might have added the present Dr. Burnett) fully developed."

The autumnal fever of Santa Maura differs from the bilious remittents that our author has seen in other parts of the Mediterranean, only in degree, and chiefly in the great debility which succeeds to the stage of excitement.

"There seems to be something in the remote cause, exclusive of the violent reaction of the system, calculated to produce this alarming state, which no tonics or stimulants can support or overcome; for, it occasionally takes place where but little febrile action has preceded it. In individuals where this direct impression has been made, and its effects are afterwards to be evolved, bleeding is a highly dangerous operation, and is generally to be avoided: the remedy is extremely unpopular, and particularly so when the season is advanced; the middle of July may be looked upon as the period at which this revolution takes place. The fever, when attended with this debility, has been miscalled typhus†; by the medical people of the country it is often called, amphimerina paludosa; but more commonly, terzana; sometimes, hemitriteo, according as they fancy that they have noted the duration and return of paroxysms or exacerbations. The medical practitioners here use emetics at first, and

* Mr. Goodisson's Historical and Topographical Essay on the Ionian Islands, 8vo. 1822.—Underwood.

† "I never knew an instance of its being communicated by contagion. In most of the hospitals on this station, the cases of fever are necessarily mixed with other diseases, from the great proportion of the former and the want of room; I never saw a case of remittent fever caught from another in hospital."

a preparation of antimony, kermes' mineral; they use the bark in large quantities. The fever, as it attacks the British in the island, is ushered in by slight shiverings, or rather fits of chilliness, alternating with transient flushes of heat; the more ardent the fever the less observable however are the chills; the headach and heat of skin then come on at once; the former is sometimes so violent as to affect the sight: I have seen it produce hemeralopia, in two instances. The affection of vision is sympathetic, and its cause, as well as that of the headach, mostly referrible to foulness of stomach. A smart emetic is here the most useful remedy, although during its operation the pain of head be sometimes aggravated by it; when, a blister applied to the nape of the neck will remove the pain altogether in many cases: a powder of twenty grains of ipecacuanha and one grain of tartar emetic, repeated in a quarter of an hour, if the first powder fail, generally succeeds in evacuating a quantity of green bile, which will also pass off sometimes by stool. This usually relieves the head after one or two hours, and procures comfortable sleep, particularly when given in the evening*. The same effect however often follows, although not a particle of bile be dislodged, simply from the shock of the emetic upon the system†. The emetic should be followed up by a purgative, when necessary, the bolus of calomel and jalap, succeeded by a dose of neutral salts, is perhaps the best. A combination of calomel and antimonial powder, varying the quantity of each according to the state of the stomach, bowels, and skin, will be found to be a most useful auxiliary to bring about a remission, which succeeds the more speedily by adopting these means, when the bark should be given without delay. The periods of exacerbation and remission should be noted in each case, and the bark administered and continued, as near to the febrile accession or exacerbation as the stomach will allow. When the periodical returns or augmentations of fever are regular, half an ounce of bark, given within an hour before the time of the attack, often interrupts the disposition to its formation and protracts the fit, sometimes even prevents it altogether. The most remarkable critical days are the third and the seventh. The fever often shews a tendency to observe the periodical revolutions of a tertian: this was remarkably the case in the summer of 1818. The exacerbations mostly occur at or about noon, and the remissions always take place towards morning. A very deep yellowness of the skin often succeeds, coming on usually about the fourth day: Of eighty cases of remittent fever occurring in the garrison of Santa Maura in the summer of

---

* "Travellers in Greece should always provide themselves with a number of emetic powders as well as bark and purgative medicines; by the former, they may often cut short a fever in a few hours; by the latter, they can remove one of its exciting causes, and with bark they may support their constitution under its formation."

† "The great danger dreaded from emetics is the effect of determination to the head: I have given at least two hundred emetics in the severest head-achs in fever, and never witnessed that effect."

1817, this symptom appeared, most strongly marked, in twelve instances. The remote cause of the fever lies in the atmosphere; the noxious exhalations having abundant sources at the margin of the lake, and the skirts of the olive wood which touch the town." 232.

The post mortem appearances were a redundancy of bile distending the gall-bladder, and extravasated into the duodenum, together with turgescence of the liver. Another remarkable phenomenon "is a contraction of the colon: in nearly one hundred cases of dissection, most of which I performed myself, and have been present at the remainder, I have witnessed this diseased structure in the greater number." Whether this alteration arises from the astringent quality of the wines, or from habitual and long-continued costiveness unattended to by the careless soldier is uncertain. How far the natives are subject to this, it is difficult to say, as the *sectio cadaveris* is seldom or never performed by them.

During the prevalence of the remittent fever and the operation of the marsh effluvium, all the other diseases are modified thereby. Thus wounds and contusions, at these periods, are often attended with erysipelatous inflammation—ophthalmiæ are exceedingly troublesome, and often require bark for the cure. Pneumonia, although prevalent to a great extent, and very unscientifically treated by the native practitioners, does not often terminate in phthisis, "a disease which is comparatively rare here." Our author avers that he has seen cases of phthisis recover here, which in other places would have been lost.

---

21. *Case of successful Bronchotomy, for the removal of a Pebble from the Trachea. By Dr. William Hunt of Dartmouth.** The patient was a boy four years of age, who, having several pebbles in his mouth, drew one of them into the rima glottidis, where it stuck fast, nearly occasioning suffocation. A lady who was present, in the attempt to remove the stone, pushed it down the trachea, and thus gave the boy the means of breathing freely. In this situation Dr. Hunt found him. He was tranquil till ordered to cough, when the pebble came up to the larynx, and occasioned convulsions with sense of suffocation, and wheezing and rattling in the throat. The stone being small, it fell back into the trachea whenever the coughing ceased. On the succeeding day an operation was agreed upon.

"The child being laid on a table with his head hanging over it, and being firmly held by the assistants, I made an incision through the integuments, two inches and a half long, near the prominence of the thyroid cartilage. On dissecting down to the trachea, a considerable vessel was opened, which required the application of a ligature.

"An incision, very little more than half an inch long, was made in the trachea, beginning from the first ring under the cricoid carti-

* Med. Chir. Trans. vol. xii.

lage, when I most fortunately felt the pebble with the point of the knife, having fixed it with the fore finger of my left hand to prevent its being drawn out of reach by an inspiration. Mr. Budland, one of the attending surgeons, with a pair of forceps easily extracted it; it was of the shape of a kidney-bean, half an inch long, $\frac{3}{8}$ of an inch wide, and a quarter of an inch thick." 29.

Violent pulmonic and tracheal inflammation ensued, but it was met by decisive measures, and the child recovered, much to the credit of Dr. Hunt and the other medical officers concerned.

Mr. Earle has appended some judicious observations on the foregoing and analogous cases, to which we would direct the attention of the surgical reader.

As the chemical details do not appear very interesting to us, or likely to bear on any practical or useful subject, we must be excused for entering upon them in this Journal.

---

22. *Small Pox and Chicken Pox.** In respect to the pathology of small pox, the few examinations which Mr. Jackson has made, shewed that the head bore the greatest marks of disease. He thinks that this might be expected from the symptoms. At an early period of the complaint there is agonizing head-ache, intolerance of light, and a sudden starting from sleep—in the latter stages there is a gradual approach to stupor.

In October and November, 1819, our author had several opportunities of inspecting the bodies after death, and, in every instance, found effusion into the ventricles of the brain—the cerebral membranes thickened and highly vascular. The subjects were under six years of age, and the small pox was of the confluent kind.

The principal object, however, of Mr. Jackson's paper is to offer further experience "tending to disprove the identity of chicken pox and modified small pox." In December 1821, Mr. Jackson's attention was directed to an eruptive disease, corresponding with what he had been in the habit of designating as chicken pox. From the most attentive investigation, it appeared evident that this eruptive disease originated in Albion-row about the middle of November—"that it there affected, indiscriminately, the vaccinated, those that had had small pox, and others unprotected—and what is very remarkable, in all those subjects the constitutional and cuticular affections were similar in degree, duration, and appearance."

"The following is the general description of the disease: considerable febrile excitement of one, but generally two days' duration, after which there was a sudden appearance of papulæ, which almost immediately became vesicular. The vesicles appeared solitary and in clusters, and where surrounded by an erythematous efflorescence

---

* Mr. Jackson, Med. Repos. No. 113.

irregularly diffused; and they arose successively during the continuance of three, four, or five days. This disease corresponded with the third species of varicella described by Willan. The vesicles were large, and their base not exactly circular; and some of them, in the progress of the disease, became pustular, or, at least, changed their colour to a light yellow; others shrivelled after the evacuation of their contents. The fourth and fifth days from its appearance, the eruption began to decline; or rather, those vesicles that had first come out then commenced shrivelling; so that in their progress towards the height, as well as on their subsidence, various states of advancement were visible."

There were no cases of small pox in the neighbourhood at the time. The disease ranged over an extent of about one mile, and not fewer than two hundred children were affected by it, of which about 50 were seen by our author.

"It may be contended by the advocates for the identity of chicken-pox and small-pox, that the disease I have described was vesicular small-pox—indeed, this is an inevitable conclusion at which they must arrive; but in what plausible way would they account for the uniform aberration, in this instance, from the usual course of disease, since in many cases (perhaps one-third of the number) there appears to have been no assignable modifying influence? Here we observe all indiscriminately affected in the same way; an eruption is displayed, differing much more widely from the character of genuine variola than this latter does from what is very properly termed modified small-pox. When small-pox appears in a vaccinated individual, its character is confessedly changed, and, unlike the disease above detailed, it seems to be as efficient as the unmodified disease in originating the true small-pox in the unprotected, or in giving rise to a disease similar to itself in the vaccinated, either by contagion or inoculation.

"Having repeatedly attempted to communicate the genuine varicella, by inoculation, to those who were unprotected by vaccination or small-pox, and having as often failed, I cannot but regard the circumstance as strongly corroborating the opinion of a specific difference in the two diseases. If small-pox, under all its usual modifications, be communicable by inoculation, and varicella not so, although the fact (if esteemed such) constitutes but an indirect proof of their non-identity, it goes far enough, I think, to induce us to infer an inherent difference in their nature."

---

23. *Rheumatic Metastasis.** We have long been convinced, from attentive observation, that the system of detracting large quantities of blood in cases of acute rheumatism, is productive of more fre-

* Mr. Armstrong, Med. and Phys. Journal, No. 289.

quent metastasis from the extremities to internal organs than a more moderate mode of treatment. In the first place, acute rheumatism is not to be controlled by large and decisive bleedings like common inflammations in other strictures, as the lungs, &c. And in the second place, if we do quell the external inflammation suddenly by copious depletion, a retrocession to some weakened organ is but too apt to take place. Of this we have seen several instances. In the following case, however, the disease was extended rather than translated to the heart, as the latter organ became affected before the rheumatism left the joints. Indeed it appears that the disease kept possession of the original seat till the last.

On the 7th November, a private soldier, young, slender form, and delicate complexion, was admitted into the regimental hospital of the foot-guards, labouring under acute rheumatism of the superior extremities, characterized by the usual symptoms, as violent pyrexia, full hard pulse, great thirst, furred tongue, and constipated bowels. Twenty-four ounces of blood from the arm in a full stream—a brisk cathartic—fever diet. 8th. No alleviation of symptoms. Again bled to twenty-four ounces, and a quarter of a grain of tartarized antimony to be taken every three hours. 9th. Symptoms a little milder, but still urgent. The antimonial continued, and 12 grains of Dover's powder to be taken at bed time. 10th. Cough—the pain in the joints, and the fever being still undiminished. A brisk cathartic of calomel and colocynth. 11th. To the cough was added pain in the region of the heart. He was again bled, and the tartarized antimony continued. 12th. The heart and arteries evinced great action, and 40 ounces of blood were abstracted, without producing syncope or making much impression on the pulse. Antimonials continued. 13th. Cough and pain in the region of the heart increased. Digitalis added to the antimony. On the evening of this day, for the first time, thirty-six leeches were applied to the region of the heart, and the digitalis increased. 14th. He died. On dissection the heart was found enlarged in size, and the surface every where covered with coagulable lymph.

We would hazard a remark or two on the above case. We think local bleeding was not employed early enough when the thoracic inflammation became developed, and we do not observe that blisters were employed at all. In the second place we conceive, (from witnessing such things on similar occasions) that there was time to introduce mercury sufficiently into the system, after the sanguineous depletion, so as to have arrested the inflammation not only of the joints but of the heart.

---

24. *Cubebs in Gonorrhœa.** The power of this medicine over gonorrhœal inflammation and secretion is not yet sufficiently ascertained. Being a new remedy it is over-rated by some, and under-

---

* S. D. Broughton, Esq. Med. Chir. Trans. vol. xii. part 1.

rated by others—it is therefore necessary to hear the experience of the neutral class. Mr. Broughton, who is a gentleman of zeal and discrimination, confines himself in this paper to a summary account of the results of fifty cases of gonorrhœa, in which the cubebs was employed. The preparations used were the powder, and the wine or tincture—the former in the doses of from half a drachm to two drachms—the latter from a drachm to half an ounce, twice or thrice a day.

"The following statement will exhibit a concise and general view of the results:—

| | |
|---|---|
| "Patients cured in from two to seven days . . . | 10 |
| - - - - - - - - eight to fourteen . . . . | 17 |
| - - - - - - - - fifteen to twenty-one . . . | 18 |
| - - - - - - - - twenty-two to thirty . . . | 1 |
| - - - - - - - in fifty-five days . . . . . . | 1 |
| Patients in whom no sensible effects were produced . | 3 |
| Total . . | 50." |

In five of the above cases, though relief was immediate and decided from the cubebs, the final cure was completed by the copaiba in four, and an astringent injection in the fifth. In one case, the complaint having been arrested by cubebs, returned, and was eventually removed by the balsam. In two cases, solely cured by the cubebs, there were swelled testicles, and in one severe chordee.

"Among *fifty* cases treated with cubebs, there were, therefore, *three* total failures, *five* relieved, and *one* suffered a relapse; leaving *forty-one* cases cured under the use of the pepper, in less time than a month, with *one* exception; the largest proportion in less than three weeks, and several in a few days, among which latter some were well in eight-and-forty or thirty-six hours; and, the failures excepted, the relief in all cases, where the symptoms were urgent, was very sudden; and only two instances of swelling of the testicles occurred, and one of chordees continuing after the exhibition of the pepper, although in other respects the clap was relieved directly.

"The account may therefore stand thus:—

| | |
|---|---|
| Cured . . . . . . . . . . | 41 |
| Relieved . . . . . . . . . | 5 |
| Cured and relapsed . . . . | 1 |
| Failed . . . . . . . . . . | 3 |
| Total . . | 50." |

Mr. Broughton is disposed to view the cubebs as, in some points, superior to all other remedies in gonorrhœa. 1st. It seems to allay irritation, and lessen the discharge better than alkalies, or the nitre and gum powders. Over bals. copaiba it holds the advantage of being admissible in the earliest and worst stages of the disease, without any inconvenience to the patient. It has not, in his experience, disordered the patient's stomach. Its superiority over injections consists in not being attended with any of the bad consequences of injections.

"As to the time which is usually consumed in removing a clap with cubebs, I think it might challenge most remedies in this respect, and is not more fickle in its operation. And, at the same time, we must take into consideration its negative qualities, which, rendering it a perfectly safe remedy, would naturally induce an inclination to sacrifice a little time, were it necessary, for the sake of avoiding the chances of a ruder, though a quicker plan of cure." 103.

Our author agrees with others, that when cubebs does not seem to act in three or four days, it should be superseded by some other remedy; or, having once relieved the urgent symptoms and its beneficial influence appearing to decline, the balsam of copaiba should be forthwith employed.

The above appears to us to be a temperate and impartial estimate of the remedy in question.

---

25. *The Knife-Eater.** Dr. Curry's history of the nautical knife-eater is as well known as Mr. Canning's story of the political knife-grinder. It was bandied about in the periodicals of the day, and has even made the "grand tour" in the Dictionnaire des Sciences Medicales.

Little imagined the honest tar, while bolting clasp-knives and swigging grog in the Bay of Biscay, that *his transactions* should descend to posterity, in company with those of the learned societies of the nineteenth century. Little did he suppose that every fragment of steel and horn which bore the marks of his gastric juice, or exhaled the odour of his appendix cæci, should be as carefully collected, preserved, and engraved, as if they had been dug out of Herculaneum, the pyramids, or the old Danish galley in the Rother!

The subject of this paper was an American sailor, who, in the year 1799, with more valour than wisdom, swallowed a clasp-knife, in imitation of a pretended exploit of the same kind performed by a French mountebank. Having found that it slipped down his throat pretty easily, especially when lubricated by some strong grog, he repeated the experiment with three other knives. In the course of the succeeding day he passed, *per anum*, three of the knives—the fourth was never evacuated to his knowledge. During the six ensuing years he swallowed no more knives; but in 1805, in consequence of a drunken bout, he swallowed fourteen knives in two days. He now paid dearly for his frolic, by pain and sickness. In Charlestown Hospital, however, he was safely delivered of his cargo of

---

* Dr. Marcet.—Med.-Chir. Trans. vol. xii. part 1.

cutlery. The next scene of operations was on board the Isis, an English 50 gun ship, at Spithead. Here he swallowed fourteen knives in the course of two days, washing them down with liberal potations of grog; and, according to the testimony of the spectators, he swallowed four more after he was fairly "sewed up," and consequently incapable of remembering the transaction! This made a sum total of at least 35 knives. He was taken ill the day after his last exploit, and came under the care of our able friend Dr. Lara, who was then surgeon of the Isis. Dr. Lara did the best he could for him; but it was three months before he passed any of the knives. Between this period and February 1807, he passed several fragments of knives, upwards and downwards, and in June of the same year he entered Guy's Hospital, under the care of Drs. Babington and Curry. He was once or twice discharged, but finally re-admitted in September 1808, and dragged out a miserable existence till March 1809, when he died in a state of extreme emaciation. With the view of dissolving the foreign bodies, or at least of blunting their asperities, Drs. Babington and Curry administered diluted mineral acids, combined with opium and mucilage. Various other palliatives were employed, with temporary benefit, but no final success.

On opening the body a black ferruginous tinge pervaded most of the abdominal organs. Some fragments were found impacted in the intestines, so as to render their exits impossible. Though some portions of knives had penetrated the coats of the colon, no fæces had escaped. On opening the stomach a great many fragments of blades, springs, and handles were found in it—some of them remarkably corroded, and greatly reduced in size—others in a state of comparative preservation.

"'The œsophagus at its lower part, and the upper orifice of the stomach, were thicker than natural. The left extremity of the stomach, where the spleen adheres to it, had its usual texture: but the right was exceedingly thickened. The rugæ, in the mucous membrane, were unusually prominent; and there were granulated projections from the edges of the rugæ. This membrane was still slightly coloured by the steel. The pylorus was natural, but the duodenum had a greater thickness than usual.'" 61.

Dr. Marcet having been present at the dissection, was enabled to notice a curious chemical fact, shewing the power which iron possesses of impregnating the biliary secretion.

"Observing that the contents of the gall-bladder partook of the black tinge of the other abdominal viscera, I collected some of the bile, for the purpose of ascertaining whether, and in what proportion, it might contain iron. About 150 grs. of this bile (which was perfectly black, and possessed the usual alkaline properties) being subjected to evaporation, and the dry mass burnt in a platina crucible, with a little wax, the incinerated residue weighed nearly five grains, and on presenting a magnet, ferruginous particles were immediately attracted by it. This residue being treated with muriatic acid and prussiate of potash, the quantity of prussian blue formed amounted, (after being well dried) to 0.5. grs. The presence of a notable quantity of iron in this bile, was therefore clearly shown by this analysis; and that this quantity was much more considerable than it is under ordinary circumstances, was ascertained by a comparative experiment upon healthy human bile (obtained from a body in which that secretion had suffered no morbid change before death,) 150 grs. of which, treated in a similar manner, yielded at most 0.2 grs. of prussiate of iron. This susceptibility of the bile, of receiving a ferruginous impregnation, from the presence of iron in the stomach, appears to me the more remarkable, as I tried in vain, a few years ago, to detect iron in the urine of persons whose digesting system was under the influence of that metal." 63.

We remember that Dr. Marcet, on the night this paper was read at the Medico-Chirurgical Society, pointed out the specimens of corroded materials as proofs of the astonishing solvent power of the gastric juice. But it was well observed, (we think, by Dr. Birckbeck,) that, considering the dyspepsy and other gastric derangements under which the patient had so long laboured, we had reason to suppose that the acetous *acid* of the stomach might contribute materially to the corrosion of the knives. This suggestion was so reasonable that Dr. Marcet did not reply to it.

The history of the case, the morbid stomach, and the *debris* of the extraneous bodies, are very well deserving of a place in the museum of the anatomist, or such a department of a work as that denominated "cas rares" in the great Dictionnaire des Sciences Medicales; but we greatly question the propriety of their introduction (after being so often published before) in the practical transactions of a medical society.

---

26. *Abdominal Tumour.** Tumours in the abdomen often puzzle the most able anatomists—the most experienced pathologists. The more facts and post-mortem examinations we can collect, the more capable shall we be of giving a probable prognosis in such cases.

A man, 31 years of age, was admitted into the St. Giles's Infirmary, in October 1822, having a tumour of considerable magnitude in the right side of the abdomen, attended with pain and vomiting, extreme emaciation, and hectic symptoms. He had been only five months ill, his first complaints being pain about the navel, with vomiting of dark-coloured substances, and black alvine discharges. In a month he discovered a small tumour in the right side of the abdomen, which gradually increased, together with the other symptoms. After his admission, the size of the tumour progressively advanced, and eventually occupied nearly the whole of the umbilical, iliac, and hypogastric regions. It was extremely hard, and somewhat irre-

---

* Mr. Ward.—Med. Repos. No. 112.

gular, but unattended with pain on pressure. His strength and flesh declined, and about three weeks before dissolution he was harrassed with a distressing diarrhœa.

" *Dissection.* The peritoneum lining the abdominal muscles was found remarkably thickened (the thickening varying from one and a half to two inches) by a substance somewhat resembling fat, but much more firm. On exposing the intestines, an appearance presented itself which at first seemed difficult to unravel: on more minute examination, however, it was evident that the tumour was formed by the same thickening or organized deposition already described, and which extended more or less over the *whole surface* of the peritoneum. The parts more immediately forming the tumour, were the lower portion of the ileum and ascending colon, which, having become agglutinated by coagulated lymph, formed an immense and compact mass. On the anterior part of this tumour was an opening formed by ulceration, through which a large quantity of matter had escaped into the cavity of the abdomen, which, in all probability, was the immediate cause of death. On following the course of the outer opening, a cavity was laid open, occupying its central part, and extending about eight inches from side to side, through which all excremental matter must have passed for some time previous to death, the structure of the intestines being at this part totally destroyed by ulceration; consequently, there were four openings communicating with the cavity formed by the ascending colon on one side, and the inferior part of the ileum on the other. The intestines through the greater part of their course were considerably constricted. The ureter and pelvis of the right kidney were greatly distended with urine. The lungs were free from tubercles, and appeared perfectly healthy. The remaining viscera were natural."

The above case, in its early stage, was mistaken for aneurism—a mistake which we have often seen committed.

---

27. *Arsenic.** A woman was supposed to have swallowed half an ounce of arsenic while in a state of pregnancy. Great sickness took place. Mr. Edwards was sent for, but how long after the accident is not stated. He found the patient much exhausted by violent retching and vomiting, complaining of cold extremities, unquenchable thirst, spasmodic pains in the bowels, parched mouth, bloated eyes, swollen face, great anxiety. The pulse was 120. Mr. Edwards lost no time in administering the carbonate of magnesia, " as an antidote to the poison," and followed the plan recommended by Mr. Hume of Long Acre. The prescription was an ounce of car-

* Mr. Edwards.—Med. and Phys. Journal, p. 288.

bonate of magnesia, half a drachm of vinum opii, half an ounce of sugar, and a pint of distilled water, of which a wine-glassful was ordered every fifteen minutes, accompanied by a free use of mucilaginous drinks. A few hours afterwards, the symptoms being rather exasperated, twenty ounces of blood were taken from the arm, and the mixture was repeated. In the evening, the patient being rather better, a dose of castor oil was exhibited, and a dozen of leeches applied to the right side, where she complained of pain. She recovered, but very slowly, and not without experiencing for some months many distressing symptoms, as indistinct vision, gastric irritability, numbness in the lower extremities, great weakness, &c.

We are of opinion that Mr. Edwards had only to combat the *consequences* of arsenic *having been* in the stomach, and that therefore the magnesia mixture, as an antidote to poison actually present, was rather nugatory.

---

28. *Mercury in Acute Diseases.* It has been very erroneously considered that the free exhibition of mercury in fevers, dysenteries, and acute inflammations, is a new and dangerous practice, principally derived from the medical men of hot climates. In the 7th volume of the "Medical Facts and Observations," there is a communication from Dr. Wright, dated 1794, shewing that the practice had been common with him and others ever since the year 1760—that is, 60 years ago. He shews that Dr. Smith, a physician at Savannha le Mar, was in the habit of giving calomel *in scruple doses* in acute diseases, with great success. In respect to dysenteries, Dr. Wright's words are these.

"In dysenteries where the fever has been considerable, the tongue dry and parched, the gripes severe, and the stools very frequent, with scarcely any thing else than blood and mucus, I have prescribed, with good effect, calomel in doses of five grains every six hours, till a copious stool or two have been procured; and afterwards in smaller doses, with occasional opiates, while the fever and gripes have continued."

Extended experience in various climates has confirmed the practice of Dr. Wright, while a correct pathology of the disease has shewn the propriety of local and general bleeding, to secure the mucous membrane of the intestines from the disorganizing effects of inflammation.

---

29. *Cancer (Retrospective.*)* Dr. Akenside did not confine his attention entirely to "the pleasures of imagination," though he was far more distinguished by his poetry than his physic. When phy-

---

* Dr. Akenside, Physician to St. Thomas's Hospital, in 1760.—Transactions of the College, vol. 1.

sician to St. Thomas's Hospital, he made some observations on scirrhus and cancer, in which he experimented on the oxymurias hydrargyri, and also on the cicuta, which was then coming into vogue for the cure of strumous affections. We shall only glance at one or two cases. The first was a woman, 50 years of age, who came under our author's care in St. Thomas's Hospital, for a scirrhous swelling on the right side of her neck, which impeded deglutition, and caused great pain in her throat and mouth. The external surface of the tumour was also very painful, and from it stinging or lancinating pains radiated over the temple, and in different directions. Dr. Akenside gave her twice a day a quarter of a grain of the oxymuriate in a spoonful of proof spirit, keeping the bowels open with some aperient waters. Under this regimen she found daily benefit. The pains gradually vanished—the scirrhous tumour diminished—the deglutition became easy—and the mouth free from soreness. On laying aside her medicines the disorder returned, and she was re-admitted as bad as ever. A repetition of the same treatment restored her once more, and she was discharged cured.

Another case of scirrhous and cancerous tongue cured by the same remedy, is related.

We do not say that the above were cases of real scirrhus or cancer; but we have seen several cases where tumours of a very suspicious appearance were dispersed by quietude, open bowels, low diet, and small doses of the oxymurias hydrargyri.

---

30. *Products of Inflammation.** Animal chemistry, we fear, is but in its infancy yet; and therefore it is not very safe to draw general conclusions till farther advances are made in the science.

It is the object of the author of the paper before us to draw a distinction between what is termed "coagulable lymph," and what is denominated "fibrin"—or, at least, to prove that it is the *latter* substance in conjunction with serum, which is deposited during the process of adhesive inflammation. Albumen cannot, he observes, be made to assume the fibrous or filamentous texture, although it be rendered solid by heat, acids, alcohol, or the galvanic fluid. When coagulated into a mass, it is easily broken down between the fingers into a pulpy form—fibrin, on the contrary, will resist, considerable pressure, being firm and elastic. "But the principal distinction between these animal products consists in the fibrin becoming solid spontaneously, when taken from the living vessels, whereas albumen has not this property."

"When adhesive inflammation is taking place, the fibrin of the blood, together with serum, seem to be secreted from the vessels; they are both thrown out in the fluid state; but the former has the

---

* T. Dowler, Esq.—Med.-Chir. Trans. vol. xii.

property of becoming solid soon after it is removed from the influence of those vessels; and in so doing it encloses between its fibres the serum, with which it had been secreted. This change begins to take place very soon after its escape from the circulating system, and does not appear to cease, till a considerable time after it has been removed from the body.

"If a blister be applied to the surface of the skin, so as to produce much inflammation, it will occasion a greater or less quantity of adhesive matter to be secreted under it; this will be generally found at the most depending part, clearly proving that it had been thrown out in the fluid form, and had arrived at this situation by its gravitation. Here it gradually assumes the solid state, and during this change it retains between its fibres, (which appear to be flattened and extended,) the serum, which is poured out at the same time; and it not unfrequently happens that the fibrin is so abundant, that the whole of the serum is enclosed in it, and that they form together one solid and opaque mass. If, however, the quantity of fibrin is but small, the solid mass so produced will be semi-transparent, and have a gelatinous appearance; hence it is usually denominated a 'gelly blister.'" 88.

If the solid compound, Mr. Dowler observes, just alluded to, be removed from the body, and pressed *strongly* in a fine linen cloth, the serum will be forced out, while the fibrous part remains:—this fibrin Mr. D. has chemically examined and compared with that of the blood, and he could not find the slightest difference between them. The solid substance thrown out by a blister, Mr. D. remarks, bears a great resemblance to the buffy coat of the blood—the nature of which seems not to have been well understood. Deyeux, Fourcroy, Thomson, all give different and unsatisfactory explanations of the phenomenon exhibited by the blood in inflammation. The following was one method which our author had recourse to for the purpose in question.

"Eight ounces of blood were taken from a man who was labouring under rheumatism, and received into two four-ounce vessels; the one being allowed to rest, in order that a buffy coat might form; while the blood in the other was kept in motion, to prevent its formation. After the lapse of twenty-four hours, the blood that had been kept agitated was washed, in order to separate the fibrin, which, when dried, weighed 12 grains.* The four ounces contained in the other vessel, being washed, and the fibrin collected and dried by the same means (having, however, the buff previously removed,) afforded only six grains; consequently, it might reasonably be supposed that the six grains wanting were existing, in some form or other, in the buffy crust. This was found to be the case; for, when it was submitted to pressure, a large quantity of fluid escaped; the mass be-

* "The proportion of fibrin varies very considerably in the blood of different individuals, and in the same person at different times."

came evidently of a fibrous structure; and, when dried, it weighed precisely six grains, making, together with the six which were obtained from the crassamentum of this blood, a number equivalent to that produced by the blood in the other vessel. The fluid thus pressed from the buff became solid by the application of heat, and consisted of common serum." 91.

From these and other experiments, our author is led to conclude that the inflammatory crust of blood is neither the altered fibrin of Deyeux, the coagulated albumen of Fourcroy, nor the coagulable lymph of Dr. Thompson—but that it is composed of "a tissue of fibrin containing between its fibres a very large proportion of fluid serum."

So far so good—but when Mr. Dowler goes on to an explanation of the *modus operandi* of Nature, in throwing out the different products of inflammation, as serum, albumen, fibrin, pus, blood, &c. we fear he "travels beyond the record," and that in a terra incognita, where we do not deem it prudent to follow him.

---

31. *Dislocations.** This work, as we expected, has had a most rapid sale, the second edition being already out of print. To this second edition, Sir Astley has given an Appendix, containing some additional matter which he considers to be worthy of publication. This plan deserves imitation by other authors, as it enables purchasers, at a trifling expence, to obtain possession of the labours of the writer, without the necessity of procuring a new edition; while it affords an opportunity to the author to add to, or subtract from, his former observations, whatever he may have omitted on the one hand, or found reason to alter on the other.

The Appendix contains three cases of dislocation of the thigh. The first occurred in Guy's Hospital, and the particulars are ably detailed by Mr. Key, Assistant Surgeon of the Hospital. It was a dislocation in the foramen ovale, and it was reduced by the mode of extension, recommended in the treatise on dislocations, excepting that the dislocated limb was carried behind, instead of before, the other.

The second case happened in St. Thomas's Hospital, and was reduced by Mr. Tyrrell, Surgeon of the Hospital. The dislocation was of the os femoris upon the pubes.

The third case occurred in the practice of Mr. Maurice, Surgeon at Marlborough, and the bone was dislocated upon the dorsum ilii. It was reduced in ten minutes by the use of the pullies and by bleeding to thirty ounces from the arm.

The next subject in the Appendix is on *fracture of the neck of the thigh bone.* It has been most falsely stated that our author had said in his work, that the union of the fracture of the neck of the thigh bone was impossible. Whereas our readers will see in our review,

---

* Appendix to Sir Astley Cooper's Work on Dislocations.

(No. 11, page 633) that he has expressly observed that, although it is a general law, that fracture does not unite, yet he denies not its possibility. He says, page 127 of the first edition—"*To deny its possibility and to maintain that no exception to the general rule can take place would be presumptuous—especially when I consider the varieties of direction in which a fracture may occur, and the degree of violence by which it may be produced; as for example, where the fracture is through the head of the bone, and there is no separation of the fractured ends; or when the bone is broken without its periosteum, the reflected ligament which covers its neck being torn; or when it is broken obliquely, partly within and partly externally to the capsular ligament; but all I wish to be understood to say is, that if it ever does happen, it is an extremely rare occurrence, and that I have not yet met with a single example of it.*"

In the Appendix, our author observes, upon this subject, that he has stated it in his work to be *a general principle*, that the neck of the thigh bone, when broken within the capsular ligament, does not unite by bone, and he has taken the trouble to learn the number of specimens in different collections of ununited fractures of the cervix emoris, and the following is the result.

| | | |
|---|---|---|
| In the collection of St. Thomas's Hospital . . | 7 | Specimens. |
| In the College of Surgeons . . . . . . . | 1 | Ditto. |
| In St. Bartholomew's . . . . . . . . . . | 6 | Ditto. |
| At Dublin . . . . . . . . . . . . . | 12 | Ditto. |
| In Mr. Langstaffe's collection. . . . . . . | 6 | Ditto. |
| In Messrs. Bell and Shaw's . . . . . . . | 6 | Ditto. |
| In Mr. Brookes's Museum . . . . . . . | 2 | Ditto. |
| In Dr. Munro's . . . . . . . . . . | 2 | Ditto. |
| In Mr. Mayo's collection . . . . . . | 1 | Ditto. |
| Total— | 43 | |

Thus, then, forty-three cases of non-union may be at any time witnessed or examined by all those who are anxious to learn the truth upon this subject; and, after this, surely no one in his senses would deny that non-union is the general result of these accidents. The main principle upon which this want of union depends, he has endeavoured to shew, exists in the lessened nutrition of the head of the bone, from the ligamentum teres being the only source of supply of blood to the detached bone after the fracture—and therefore it scarcely undergoes any ossific change.

But the circumstance which strikes us as being most decisive upon this subject, is a preparation which our author states to be in the possession of Mr. Langstaffe, in which there is a fracture *within* and another *external* to the capsular ligament. Now we would ask what ought to be the result of such a case, if the principle advocated by our author be not well founded?—Certainly both fractures ought to be united, or both ununited. But what is the fact?—That fracture which is *external* to the capsular ligament "is firmly united;" while that which is situated *within* the ligament, "has undergone no ossific change." These fractures must have been situated within three inches of each other, and the means which succeeded with the one should have been successful in the other. Still, however, our author does not object to the surgeon, (" if he has the smallest doubt either of the nature of the accident or of its result") employing the means which may suggest themselves to his mind as holding out the hope of a successful issue.

Mr. Stanley, Assistant Surgeon of St. Bartholomew's Hospital, has kindly shewn our author a preparation which conveys an idea, that the neck of the thigh-bone had been broken and afterwards re-united. But a great deal of doubt must arise upon this subject so soon as it is known that—" the neck of the other thigh-bone, in the same subject, exhibited nearly similar appearances." Is it not more probable that this is merely that common change which arises from the absorption of the phosphate of lime in the neck of the thigh-bone, in old persons? We would also ask, whether or not there was any appearance of rickets or other disease in the bones of this subject?—But admitting, for a moment, that in this person both the necks of the thigh-bones were broken, and had both united—it would be so extraordinary a case as only to prove an exception to a general rule, and could never be admitted to establish or subvert a general principle.

Our author concludes this subject with observing that, in all cases in which the limb is but little shortened, and when a crepitus can be distinctly perceived upon rotating the bone, as it will not have the appearances of fracture within the capsular ligament, it will be best to treat it as a fracture external to that ligament.

Sir Astley then proceeds shortly to state the changes which the neck of the thigh-bone undergo in old and diseased persons, and he subjoins a letter from Mr. Colles, Professor of Anatomy and Surgery at Dublin, and who has most ably written upon the subject of fracture of the neck of the thigh bone. This letter we shall give at full length.

" *Stephen's Green, Feb.* 12, 1823.

" My Dear Sir,

" Since the receipt of your letter, I have carefully examined all the specimens of fractures of the neck of the thigh-bone contained in both Museums of our College of Surgeons. In that which is appropriated to the use of the School, I find seven instances of fracture within the ligament, each of these have been described in my paper cn this subject, in the Dublin Hospital Reports. Since the publication of that Essay, the Conservator of our College-Museum has collected five specimens of fracture within the ligament. In this Museum are also four instances of fracture external to the condyle ligament. In the School-Museum are two instances of fracture external to the ligament. Of this latter description of fracture, less than one half the number are united by bony union. Of the fractures within the ligament, not one has made a nearer approach to bony union than that described in the paper alluded to. I must say, that I have never yet seen an instance of bony union where the fracture had been within the ligament. We have very many specimens of a disease of the head and neck of the thigh-bone, which is of frequent occurrence amongst our labouring poor. On this subject I have some idea of writing a paper for the next volume of the Dublin Hospital Reports, and of endeavouring to shew, that in all probability the supposed cases of fracture within the ligament united by bone, were merely instances of this disease.

"If you have any wish for them, I shall have great pleasure in sending you sections of some of these cases, which I am certain might be passed upon many surgeons for fracture of the neck of the bone. "I am, my dear Sir,

"Your most sincere friend,

"A. COLLES."

The next subject in the Appendix is on *dislocation of the knee-joint*, of which a most interesting case is given from Mr. Toogood, Surgeon at Bridgewater.

*Dislocation of the Os Humeri.* A case of partial dislocation of the os humeri forwards, and a case of dislocation backwards on the dorsum scapulæ, are given under this head. For the latter, Sir Astley Cooper acknowledges himself indebted to Mr. Perry, (who dates his letter from the apartments of the House Surgeon of St. Bartholomew's Hospital) in the following terms:—"Mr. Perry, without solicitation had the kindness to send me the foregoing case, for which I am indebted to him. Our large hospitals in London should be made as conducive as possible to the public advantage, by a liberal and reciprocal communication." Now, next to labouring hard himself, nothing is so praiseworthy, in any individual, as exciting the younger persons to a zealous and industrious pursuit of their profession. We have known our author for more than twenty years, and, we have always found him inviting the younger branches of the profession to exertion, and assisting them, by all the means in his power, to attain that knowledge which would make them happy in themselves and useful to the public.

*Fracture of the Neck of the Os Humeri.* A fatal case of this accident is given from a patient, aged 72, admitted into Guy's Hospital. An interesting case of compound dislocation of the os humeri, at the elbow joint, is given as it presented itself at Guy's Hospital, and which terminated successfully. Of lateral dislocation of the radius, Mr. Freeman, Surgeon in Spring Gardens, brought our author an example. It is a very rare accident. A case is also given from Mr. Tyrrell, of dislocation of the radius forwards. Cases of fracture of the condyles of the os humeri—compound fracture of the olecranon, and compound dislocation of the wrist—ulna projected, and radius broken, conclude the appendix.

